# The history, state of the art and future prospects for oleaginous yeast research

**DOI:** 10.1186/s12934-021-01712-1

**Published:** 2021-12-07

**Authors:** Felix Abeln, Christopher J. Chuck

**Affiliations:** 1 Department of Chemical Engineering, University of Bath, Bath, BA2 7AY UK; 2 Centre for Sustainable and Circular Technologies, University of Bath, Bath, BA2 7AY UK

**Keywords:** Biodiesel, Climate change, Triglyceride, Industrial biotechnology, Microbial lipid, Oleaginous yeast, Single-cell oil, Sustainability

## Abstract

Lipid-based biofuels, such as biodiesel and hydroprocessed esters, are a central part of the global initiative to reduce the environmental impact of the transport sector. The vast majority of production is currently from first-generation feedstocks, such as rapeseed oil, and waste cooking oils. However, the increased exploitation of soybean oil and palm oil has led to vast deforestation, smog emissions and heavily impacted on biodiversity in tropical regions. One promising alternative, potentially capable of meeting future demand sustainably, are oleaginous yeasts. Despite being known about for 143 years, there has been an increasing effort in the last decade to develop a viable industrial system, with currently around 100 research papers published annually. In the academic literature, approximately 160 native yeasts have been reported to produce over 20% of their dry weight in a glyceride-rich oil. The most intensively studied oleaginous yeast have been *Cutaneotrichosporon oleaginosus* (20% of publications), *Rhodotorula toruloides* (19%) and *Yarrowia lipolytica* (19%). Oleaginous yeasts have been primarily grown on single saccharides (60%), hydrolysates (26%) or glycerol (19%), and mainly on the mL scale (66%). Process development and genetic modification (7%) have been applied to alter yeast performance and the lipids, towards the production of biofuels (77%), food/supplements (24%), oleochemicals (19%) or animal feed (3%). Despite over a century of research and the recent application of advanced genetic engineering techniques, the industrial production of an economically viable commodity oil substitute remains elusive. This is mainly due to the estimated high production cost, however, over the course of the twenty-first century where climate change will drastically change global food supply networks and direct governmental action will likely be levied at more destructive crops, yeast lipids offer a flexible platform for localised, sustainable lipid production. Based on data from the large majority of oleaginous yeast academic publications, this review is a guide through the history of oleaginous yeast research, an assessment of the best growth and lipid production achieved to date, the various strategies employed towards industrial production and importantly, a critical discussion about what needs to be built on this huge body of work to make producing a yeast-derived, more sustainable, glyceride oil a commercial reality.

**Supplementary Information:**

The online version contains supplementary material available at 10.1186/s12934-021-01712-1.

## Introduction

All microorganisms are composed of lipids usually comprising around 6 to 8% (w/w) of their dry cell weight [1]. Certain microorganisms including eukaryotic (moulds, yeast, algae) and prokariotic (bacteria) species intracellularly produce larger amounts of lipids, in the form of particles (also termed droplets or bodies), typically for carbon/energy storage [2]. Microorganisms producing more than 20% (w/w) of their dry cell weight as lipids are termed oleaginous [3]. The microbial lipids are sometimes called single cell oils (SCOs) [4]. These lipids are not only a promising source of oil for biofuel production but for human and animal nutrition as well.

Oleaginous yeasts are often described as superior for possible commercial lipid production over other oleaginous microorganisms, due to their fast growth, high lipid content and volumetric productivity [5]. Yeast lipid processes have been developed for over a century [6] seeing more than 700 research articles published in over 180 different academic journals. However, despite several advantages over plant oils [7, 8], it was not until recently that commercial production has commenced, in the form of a speciality oil [9, 10].

Within this topic this review focuses mainly on the upstream processes and aims to address three key questions: firstly, what central efforts have been undertaken within this field; secondly, what are the most popular and promising feedstocks, organisms, operation conditions and applications for oleaginous yeasts; and thirdly, how can the knowledge of the past performance aid commercialisation of affordable yet sustainable yeast lipid processes? To facilitate addressing those questions, oleaginous yeast performance data from the majority of published research articles concerning oleaginous yeast since 1972 was extracted, analysed and interpreted. The full methodology used to collect the data is given in Additional file 1.

## Industrial development and key research for oleaginous yeasts

### Industrial development

**Fig. 1:**
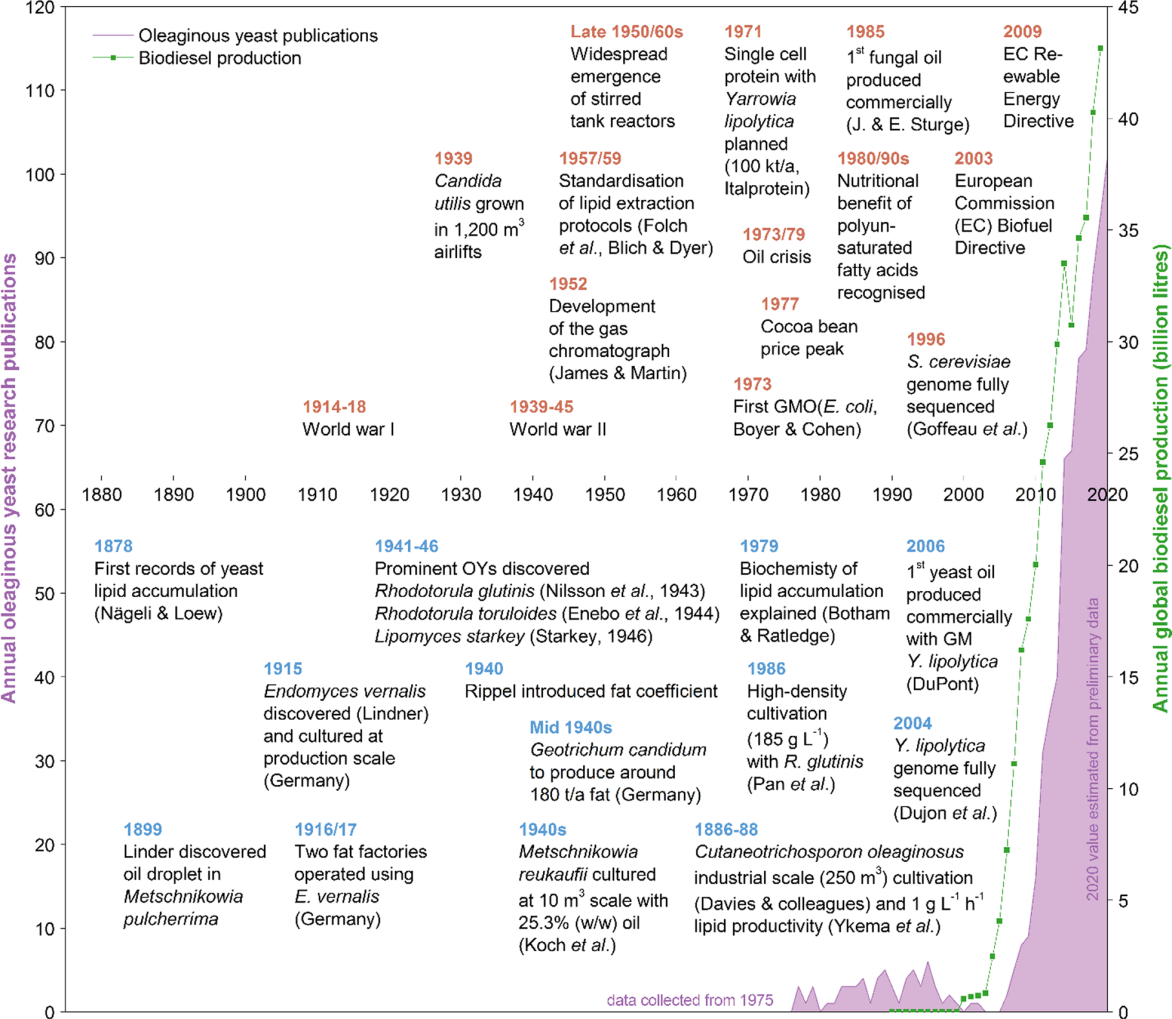
Timeline of yeast lipid research. Displayed is the annual amount of oleaginous yeast research publications since 1975 in English language together with the annual global biodiesel production (biodiesel data obtained from [254]). Key developments associated to the field are displayed in the top half of the graph (year label in brown) and those directly concerning oleaginous yeasts in the bottom half (blue). The full methodology used to collect and analyse the presented data is given in Additional file 1

Records describing the production of fats from yeast date back to 1878 [11]. In 1895, a yeast termed *Torula pulcherrima* (now *Metschnikowia pulcherrima*), was discovered producing an oil droplet [6]. However, to aid aeration the production was limited to shallow trays, far from feasible on the industrial scale [6, 12, 13]. In 1915, the fungus *Endomyces vernalis* was demonstrated to produce up to 42% (w/w) lipid under nitrogen limitation [6]. During World War I, two factories were established in Germany producing fats in trays; the yeast produced was used directly as a paste for human consumption, but ultimately issues with contamination led to closure of the facilities [12, 13]. In the interwar period, a large research effort discovered a range of new oleaginous species (Fig. 1) [14–19].

By World War II, microbial lipids were considered more as a mechanism for energy storage [20]. Accordingly, increased lipid synthesis was often observed when starving cells of a nutrient other than carbon, and reducing anaerobic pathways [6, 18, 20, 21]. Although the analysis was comparably complex at the time, researchers found that, typically, the oil was similar in composition to vegetable oils mostly containing triacyglycerols (TAGs; Additional file 1: Table S1) [17]. From 1939, a number of industrial scale processes were established including a “fat yeast” with 20% (w/w) to 30% (w/w) oil [12, 22], and *M. reukaufii* containing 25.3% (w/w) oil at the 10 m^3^ scale using molasses and whey [23]. In the last years of World War II and the years thereafter, three factories using *Geotrichum candidum* with 20% (w/w) lipid content were established in Germany, each with a capacity of around 60 tonnes fat per year, using whey and lignocellulosic hydrolysate [12].

In the decades after World War II, the emergence of stirred tank reactors (STRs) led to further advances in lipid research (Fig. 1). In this time the experimental lipid production limit of around 0.22 g g^−1^ when using sugars as carbon source was established [24]. In 1979, Botham and Ratledge published the first concise biochemical explanation of yeast lipid accumulation [25]. Meanwhile, Italproteine, a joint venture of British Petroleum (London, UK) and Azienda Nazionale Idrogenazione Combustibili (Rome, Italy), developed a process involving *Yarrowia lipolytica*, a potential oleaginous yeast, for single cell protein (SCP) production at 100,000 tonnes per year in 1971 [26]. The plant was completed by 1977, but the product was never approved for animal feed by the Italian authorities, amid concerns over the substrates used for the production.

A yeast lipid process was close to commercialisation in the 1980s to produce a cocoa butter equivalent (CBE) at 1000 tonnes per year [27]. The process was developed with *Cutaneotrichosporon oleaginosus* on whey [28–32]. The promising characteristics of this yeast made *C. oleaginosus* the most investigated species during this time (Additional file 1: Fig. S1). Lipid productivities of up to 1.0 g L^−1^ h^−1^ were achieved with this species [28]. It could produce an oil of high quality with a high conversion ratio of the lactose [33] at pilot (500 L bubble column, 8.2 m^3^ STR) and industrial scale (250 m^3^ bubble column) [27, 31]. However, with a drop of the cocoa butter price, the project was terminated due to the low profitability, competition with low-cost CBE from palm oil fractionation, and uncertainty of market acceptance [33].

In the 1990s, the rapid development of genome sequencing and genetic engineering rekindled interest in yeast lipid production, which allowed for the development of high-value yeast lipids tailored to the speciality oil market. Eight years after *Saccharomyces cerevisiae*, Dujon et al*.* fully sequenced *Y. lipolytica* [34], and in the same year, Mlíčková et al*.* [35] genetically modified *Y. lipolytica* to produce increased amounts of lipids.

As a result of these developments, commercial production of yeast lipids commenced in 2006, developed by DuPont (Wilmington, USA). The researchers genetically modified *Yarrowia lipolytica* to produce increased amounts of C20 fatty acids, mostly eicosapentaenoic acid (EPA, C20:5 [9, 10]). The yeast contained around 35% (w/w) lipid and 15% (w/w) EPA [36]. At that time, EPA was the last of the four major ‘conditionally essential’ fatty acids to be commercially produced by a microbe. The lipids were produced by CPKelco (Atlanta, USA) at the 4.5 m^3^ scale [36] and sold in the USA as NewHarvest™ EPA oil for human consumption from 2010, and the yeast itself used as animal feed for Verlasso^®^ salmon [10], in partnership with AquaChile (Puerto Montt, Chile). However, though the oil was designated Generally Recognized As Safe (GRAS) by the U.S. Food and Drug Administration (GRN000355), is was criticised by consumers for carrying traces of hexane and being produced by a genetically modified organism (GMO).

### Twenty-first century research

Particularly in the last decade, more research effort has been channelled into advancing yeast lipid technology as a sustainable source of oil to displace palm and soybean oil, mostly as a novel route to advanced biofuels. This is reflected in the yearly number of publications concerning oleaginous yeasts, which has seen an annual growth of 26.6% (Compound Annual Growth Rate, CAGR) in the last decade (2009 to 2019), compared to a growth of 3.1% in total scientific publications (Fig. 1). At least 95 oleaginous yeast research papers were published in the English language in 2019 alone, equivalent to 35.2 per million scientific publications, up from 5.2 per million in 2009. Arguably, the most influential driver for this surge was the desire to produce biofuels sustainably, largely in the form of biodiesel (Fig. 1). The European Commission, for instance, adopted the Biofuel Directive (2003/30/EC) in 2003 aiming to transition to 5.75% or above, biofuel in road transportation by 2010. The Renewable Energy Directive (RED I, 2009/28/EC) released in 2009, increased this amount to 10% by 2020 [37, 38]. Boosted by subsidies, such directives [38] were thought to have led to a sharp increase of global biodiesel production from the mid-2000s (CAGR 22.3% from 2006 to 2013; Fig. 1). Whilst prior to these developments, oleaginous yeasts have only rarely been proposed for biofuel production [39], a sharp increase in interest was seen in 2006, and since then most publications (85 ± 11%) have proposed biodiesel as an application for the produced lipids.

Promising advances have been made because of these increased efforts, pushing the boundaries of oleaginous yeast research, in terms of achieved lipid yield, content and productivity, but also in further elucidating the associated biochemistry. For instance, lipid yields around 0.25 g g^−1^ saccharide have been reported frequently through fed-batch cultivation using evolved or engineered yeast [40–42]. Due to the rapid development of the appropriate genetic tools, *Y. lipolytica* has become the most popular oleaginous yeast (Additional file 1: Fig. S1). Interestingly, it has been shown that nutrient limitation is not such a key for some native yeasts such as *M. pulcherrima* [43] and *Solicoccozyma terricola* [44, 45], increasing the feedstocks suitable for yeast lipid production without additional pretreatment, such as waste streams in the circular economy, and allowing to simultaneously enhance growth and lipid production rates [43]. This beneficial trait has also been engineered into other strains such as *Y. lipolytica* [46]. Importantly, is has been shown that lipid production can be uncoupled from biomass production through releasing lipids into the broth, for example when using acetate in combination with *C. oleaginosus* [47] or through genetic modification of *Y. lipolytica* [48]—potentially maximising lipid yields and facilitating downstream processing. Unsurprisingly, a corresponding ‘lipid content’ of 120.4% (w/w) is the highest yet reported in the academic literature [48]. The emerging techno-economic analyses (TEAs) and life-cycle assessments [49–52] further advance the credibility of the yeast lipid concept. In such analyses, high productivities are often deemed crucial for economic lipid production [49, 50]. For achieving those, fermentation at high cell densities, already obtained with *R. toruloides* in 1986 (185 g L^−1^) [53], has been increasingly popular, such as with *C. oleaginosus* (104 g L^−1^) [40], *M. pulcherrima* (122 g L^−1^) [54] or *Y. lipolytica* (116 g L^−1^) [55], with a highest lipid productivity of 1.2 g L^−1^ h^−1^ achieved in fed-batch operation using genetically modified (GM) *Y. lipolytica* [41]. It shall be emphasised however, that despite these promising advances, commercial production of a yeast-derived commodity oil currently remains elusive.

Most oleaginous yeast research has been conducted by authors associated with institutions in China (nearly 13% of publications), USA and India (Additional file 1: Fig. S2). Relative to their total scientific output however, Thailand (100 publications per million scientific publications) and Greece [56] are most active in this field, whereas USA, UK, Japan, and Germany only to a lesser extent (Additional file 1: Fig. S2). To a large extent, these statistics are attributable to the countries/unions’ policies as well as the interests of local research groups. For instance, the strong research output in Thailand from 2010 could be linked to its introduction of biodiesel blends, mandatory from 2012, largely to reduce smog, absorb palm oil surplus and support prices [38]. In terms of research groups, C. Ratledge (UK) contributed immensely to the elucidation of lipid accumulation mechanisms in the second half of the twentieth century [2, 25, 57]. Nowadays, the largest contributors to oleaginous yeast knowledge are the frequently collaborating groups around S. Papanikolaou, G. Aggelis, and A.A. Koutinas (Greece), developing processes largely with *Y. lipolytica* and *R. toruloides*; the group around J.M. Nicaud (France) focussing on the *Yarrowia* clade; and the groups around Z.K. Zhao, H. Shen and Z. Gong (China), further advancing the knowledge of *R. toruloides* and *C. oleaginosus* (Additional file 1: Fig. S3).

### Alternative heterotrophic oil production

A substantial amount of research effort has also been spent on developing heterotrophic algae, bacteria (for non-edible oils) and moulds as hosts for lipid production [58]. Heterotrophic algal oils occupy large market shares for some long-chain polyunsaturated fatty acid (PUFA) production for over two decades due to the algae’s natural ability to produce those fatty acids [59]. Arguably, the experiences in the cultivation of other fungi for lipid production had the largest impact on yeast lipid research. Indeed, the ability of mould to produce increased amounts of lipids was documented shortly before yeast (1873) [2, 60]. The advantage of mould over yeast is the natural ability to produce gamma-linolenic acid (GLA) [61, 62], but also arachidonic acid (ARA) [63], docosahexaenoic acid (DHA) [64] and EPA [65]. From 1985 to 1990, J. E. Sturge produced and sold a commercial microbial oil in the UK for human consumption, using *Mucor circinelloides* and containing increased amounts of GLA [59]. Importantly, this event demonstrated that commercialisation of certain microbial oils was possible. Indeed, commercialisation of other fungal processes producing long-chain PUFAs occurred thereafter, most of which are still produced today [33, 66]. For example, the production of an ARA-rich oil by *Mortierella alpina* has been estimated at around 9000 tonnes per year (2013) [67].

## Oleaginous yeast key knowledge

### Lipid composition in oleaginous yeasts

In oleaginous yeasts, the accumulated lipids are non-polar, primarily C13 to C21 triacyglycerols (TAGs) and steryl esters (SEs). The exact composition of the lipid strongly depends on the yeast species, but also environmental conditions such as the carbon source [68, 69]. Lipid particles of the non-oleaginous *Saccharomyces cerevisiae*, for example, contained 51.2% (w/w) TAGs, 44.4% (w/w) SEs, as well as 2.6% (w/w) proteins and 1.3% (w/w) phospholipids [70]. In contrast, for an oleaginous yeast, the primary fraction are typically TAGs, such as in *Yarrowia lipolytica*, with 84.5% (w/w) TAGs, 7.8% (w/w) SEs, 5.1% (w/w) proteins and 2.0% (w/w) phospholipids [69]. Generally, the TAG content of the lipid in oleaginous yeasts is around 80% (w/w) to 90% (w/w) in most cases [28, 29, 71–73]. The lipids extracted and analysed in experiments typically include those present in the cell membrane of the yeast, and not only the lipid droplet. The predominant fatty acids of yeast lipids are oleic (C18:1), palmitic (C16:0), linoleic (C18:2) and stearic (C18:0) acid [74].

### Fatty acid synthesis in oleaginous yeast

**Fig. 2:**
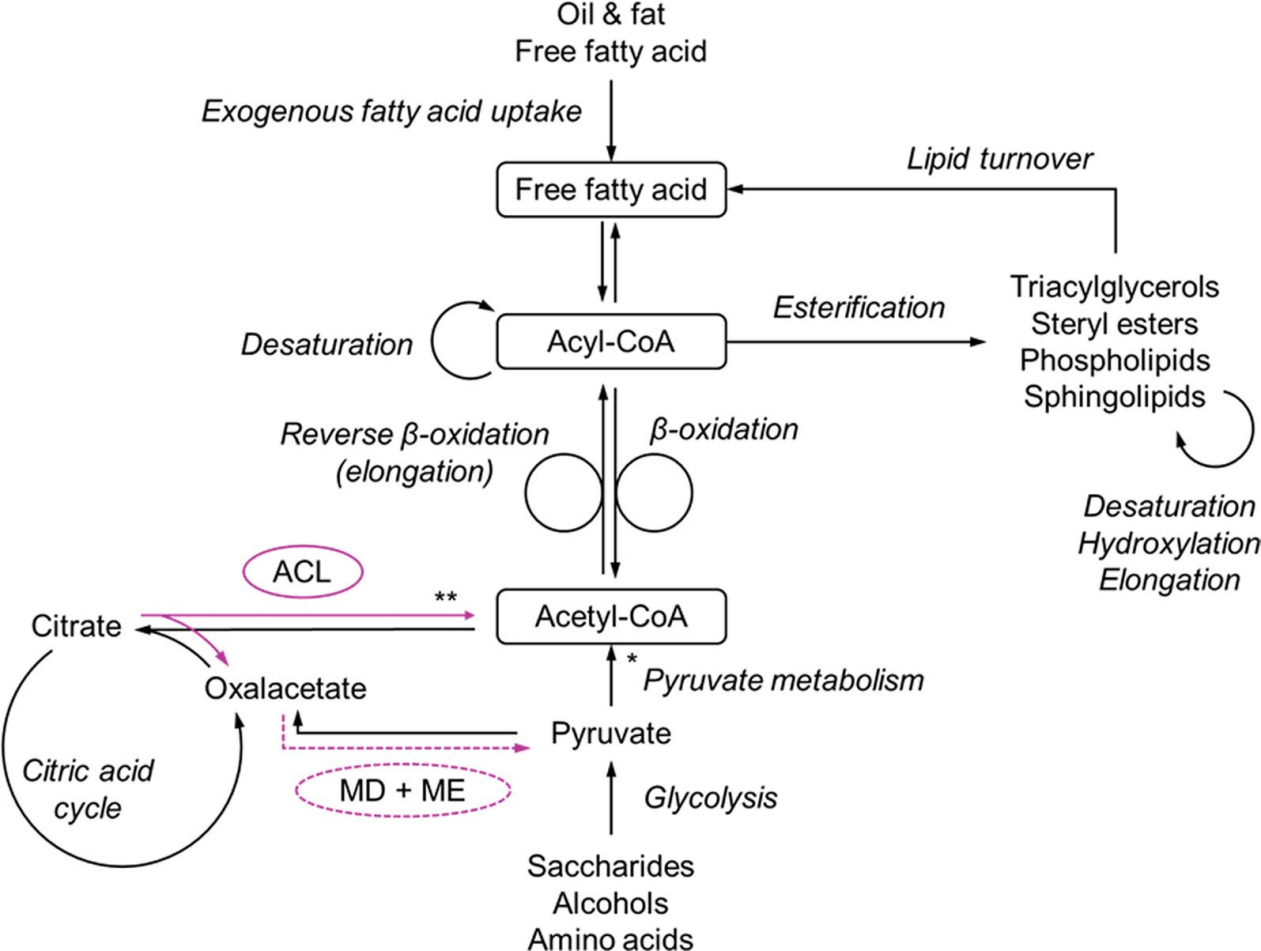
Simplified schematic description of the yeast fatty acid metabolism with a focus on de novo lipid formation. *ACL* ATP citrate lyase, *MD* malate dehydrogenase, *ME* malic enzyme; the enzymes catalyse reactions specific for oleaginous yeasts. *Pyruvate enters the mitochondria and undergoes oxidative decarboxylation to mitochondrial acetyl-CoA or is converted to cytosolic acetyl-CoA via the pyruvate–acetaldehyde–acetate pathway requiring ATP; **citrate is transported from the mitochondria into the cytosol and cleaved to cytosolic acetyl-CoA

Yeast lipids can be produced via the anabolic de novo or ex novo pathway. In the de novo pathway in non-oleaginous yeasts, the fatty acid precursor acetyl-coenzyme A (CoA) is issued by glycolysis and the pyruvate metabolism, but in oleaginous yeasts it can also be obtained from citrate, channelled from the citric acid cycle and cleaved by the enzyme adenosine triphosphate (ATP) citrate lyase [25, 75]. This enzyme is absent in non-oleaginous yeasts, which under nutrient-limiting condition typically divert excess carbon into citrate or polymers [76, 77]. Mediated by acetyl-CoA carboxylase, acetyl-CoA is then carboxylated to malonyl-CoA, both of which are supplied to the fatty acid synthesis pathway as two-carbon donors (Fig. 2).

The fatty acid synthesis, a cyclic series of condensation reactions, sometimes referred to as the reverse beta-oxidation pathway, is catalysed by fatty acid synthases and elongases, resulting primarily in saturated C16 and C18 fatty acyl-CoA [78]. The formation of unsaturated fatty acyl-CoAs is catalysed by fatty acid desaturases [75]. As these are oxygen-dependent biochemical reactions, low oxygen availability can result in a higher degree of saturation in yeast lipids [31]. The fatty acyl-CoAs are the precursors for lipid formation in yeast cells such as for storage TAGs and SEs. For TAG formation, the fatty acyl-CoAs are combined with glycerol-3-phosphate, either formed from a glycolysis intermediate or glycerol, through condensation and de-phosphorylation, yielding diacylglycerol and finally TAG. The final step, acylation, is catalysed by diacylglycerol acyltransferase. In the case of SEs, also a product of acylation, sterols are acylated instead of a diacylglycerol.

In most oleaginous yeasts, lipid accumulation is triggered by limitation of a nutrient. Excess citrate accumulated in the citric acid cycle is then channelled into fatty acid synthesis [25]. Nitrogen, phosphorus, iron, sulphur, zinc or oxygen have been limited frequently to trigger lipid accumulation [79–81]. Storage lipids may be degraded through beta-oxidation, thereby releasing the energy stored in carbon bonds, if metabolic requirements cannot be satisfied through an extracellular carbon source [82]. If sufficient nutrients are available, the intracellular lipids may be mobilised for cell proliferation [83]. The carbon/nitrogen (C/N) or carbon/phosphorus (C/P) ratio are often stated to appoint limiting conditions of the respective nutrient. A key characteristic of de novo lipid production triggered by nutrient limitation is therefore the division of cell growth and lipid accumulation for the most part. A sustained key nutrient deprivation can result in the accumulation of side products such as citrate. Previously it was assumed that oleaginous yeasts simultaneously accumulated both lipid and intracellular polysaccharides in the de novo pathway. However, more recent work has shown that some oleaginous yeasts accumulate high levels of polysaccharides under nitrogen-rich conditions, before converting these intracellularly to lipids upon nitrogen limitation [84, 85].

Important substrates and co-factors for de novo fatty acid synthesis are acetyl-CoA, ATP, nicotinamide adenine dinucleotide phosphate (NADPH), biotin and pantothenate [75, 77, 78, 86]. Whilst they can be synthesised by most yeast given an energy source is available, their supplementation to the growth medium may be crucial to achieve high yields and productivities, as they require energy to be produced and are also required by other cellular processes.

In the ex novo pathway concerning exogenous fatty acid uptake, free fatty acids, either available directly as substrate or from hydrolysis of other hydrophobic substrate through secreted or cell-bound lipases, are actively transported across the cell boundaries [77]. Inside the cell, they are further broken down into smaller chain acyl-CoAs and acetyl-CoAs, catalysed by acyl-CoA oxidases in the beta-oxidation pathway. These intermediates are used for cell growth and maintenance, as channelled into the citric acid cycle, but also used as precursors for lipid synthesis. Compared to the de novo pathway, in ex novo cell proliferation and lipid accumulation typically occur simultaneously as culture conditions are not normally appointed nutrient limiting. The ex novo synthesis route is commonly used for the upgrading of low value lipids, such as waste oils, into lipids with a far higher market value such as a substitute for cocoa butter [77].

## Feedstocks for oleaginous yeasts

**Fig. 3:**
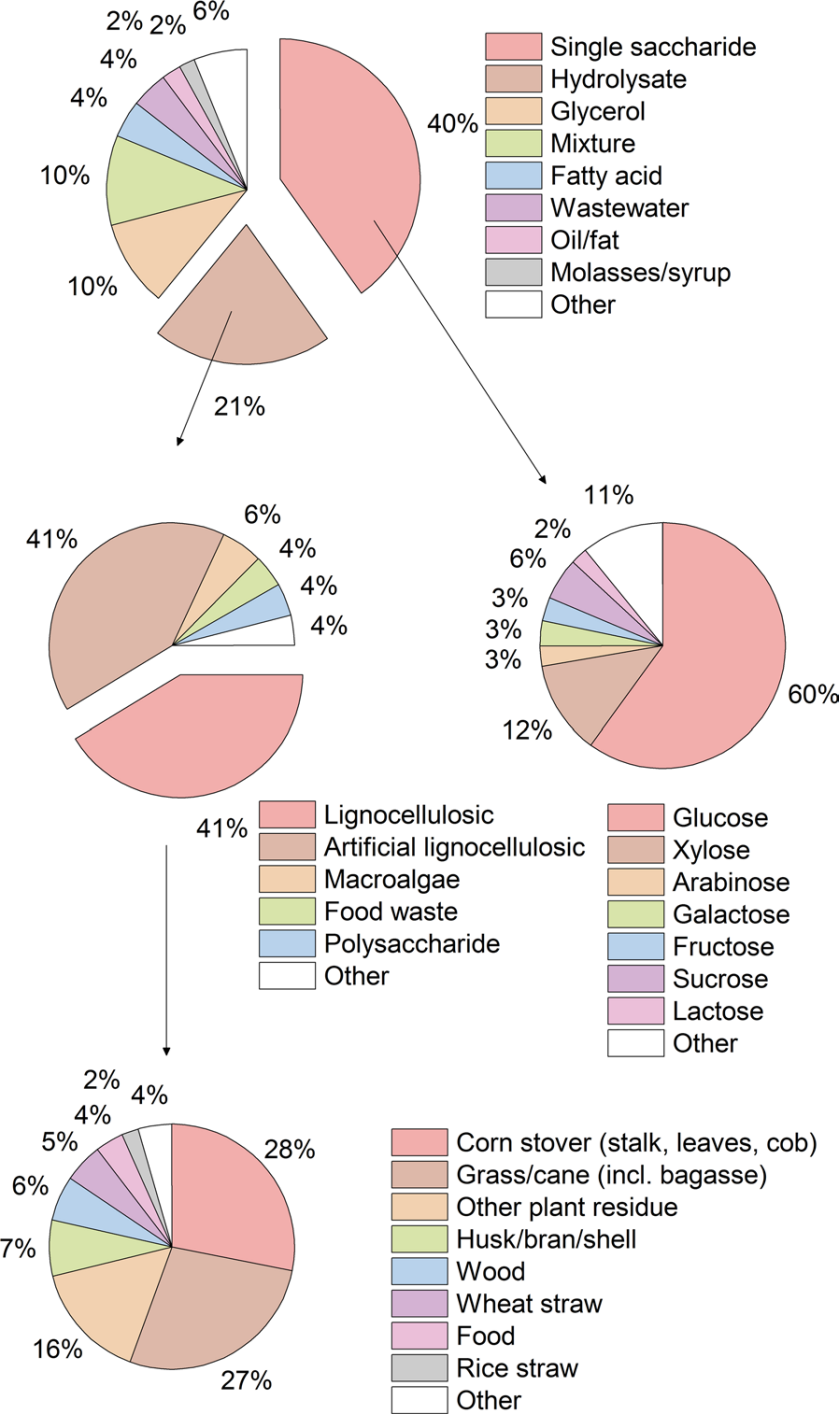
Feedstock distribution of oleaginous yeasts. Displayed are the percentages of main carbon sources used in oleaginous yeast research. Please note that sometimes multiple carbon sources are used in a single publication. The category ‘single saccharide’ includes all single saccharides, sugar acids and alcohols; ‘hydrolysate’ also artificial hydrolysates; and ‘mixture’ the mixtures of all other carbon sources. The full methodology used to collect and analyse the presented data is given in Additional file 1

A wide range of carbon sources has been used for yeast lipid production: single saccharides, hydrolysates, and glycerol (crude and pure) have been the most frequently used carbon sources, together accounting for around 71% of feedstock used (Fig. 3). Less popular have been fatty acids, wastewaters, oils/fats, and molasses/syrups; and infrequently used alcohols, aromatics, aqueous extracts, and other waste streams (Fig. 3). The ability to use a wide range of substrates is a key advantage of an oleaginous yeast and can dramatically improve the sustainability of the process aligning with specific policy directives such as the EU Renewable Energy Directive. The carbon source markedly influences the lipid synthesis [56, 69, 87–90], particularly whether sugar- or fat-based (de novo versus ex novo pathway). The feedstock is typically chosen based on suitability to an organism, whether containing a limiting nutrient, simplicity and, particularly on the industrial scale, on cost and availability [91]. Molasses, for instance, is frequently used in industrial fermentation, but the comparably high nutrient load (C/N ratio around 20 to 40 g g^−1^ [92]) makes it unsuitable for many oleaginous yeasts. For a feedstock containing excess nutrients, processing strategies such as nutrient removal have been suggested [80, 93], but the use of yeasts not requiring nutrient depletion such as *M. pulcherrima* [43], *S. terricola* [44, 45] or GM *Y. lipolytica* [46] significantly simplify the processing.

### Saccharides

Single monosaccharides have overwhelmingly been the prevalent carbon source used in oleaginous yeast research, with the majority being glucose and xylose (Fig. 3). Not surprisingly, single glucose as a carbon source accounted for nearly 25% of feedstock used and has been used in over 60% of oleaginous yeast publications (Additional file 1: Fig. S4). Xylose and arabinose, on the other hand, are often introduced to investigate the suitability of a yeast species to ferment lignocellulosic hydrolysates [94–96]. The most prevalent single disaccharides have been sucrose and lactose (Fig. 3). Often the suitability for cultivation on molasses [97, 98] and whey [99], respectively, are tested. Starch has been the most used polysaccharide, and some oleaginous yeasts possess suitable amylotic activity [100–102].

Despite the advantages, using purified saccharides as a carbon source significantly hampers the economics of the process (approx. US$ 450 per tonne glucose, 2018), hence they are unsuitable for commercial production of a lower value lipid [49, 52]. Numerous recent investigations focussed on developing alternative low-cost substrates [103].

### Hydrolysates

A suitable growth medium can be generated through the hydrolysis of organic matter [104–107]. Lignocellulosic biomass is attractive as a hydrolysis feedstock in terms of availability, and research into macroalgae has been presented to produce a food-grade oil [104]. The sugar selling price of a hydrolysate is typically lower than purified monosaccharides and has been estimated as US$ 255 per tonne (2007) for a dilute lignocellulosic hydrolysate [106]. However, the technical challenge is often to effectively use the full range of carbon sources available and reduce the impact of frequently introduced fermentation inhibitors on both biomass and lipid production, which often adversely affect the growth and lipid content [104, 105, 108].

To a large extent, lignocellulosic material, foremost corn stover, grass/cane or other plant residue, has been used for hydrolysis in oleaginous yeast research (Fig. 3). Alternatively, artificial hydrolysates have been generated to test suitability of a yeast strain to produce lipids from lignocellulosic hydrolysates. These typically include mixtures of glucose, xylose, arabinose and/or acetate, sometimes cellobiose [94–96, 109]. Together, real, and artificial lignocellulosic hydrolysates account for around 82% of hydrolysates used (Fig. 3). Native oleaginous yeasts capable of co-metabolising glucose and xylose, the major carbon sources in lignocellulosic hydrolysates, include *L. starkeyi* [110, 111], *C. oleaginosus* [96], *Pseudozyma hubeiensis* [95], *M. pulcherrima* [112]*, Trichosporon cutaneum* [94] and *Trichosporon coremiiforme* [113]. Partially hydrolysed media, used to reduce process complexity, cost or inhibitor formation, typically contain some polysaccharides [104, 114], wherefore oleaginous yeast with oligosaccharide degrading capacity, such as *C. oleaginosus* [115] or *M. pulcherrima* [104, 116, 117], have been used. To tackle fermentation inhibitors, milder hydrolysis conditions [104, 114], detoxification [105, 118], fermentation control such as through a high pH [119] or high-density continuous processing [54, 120], the use of native strains with high inhibitor tolerance such as *C. oleaginosus* [118, 121–123], *M. pulcherrima* [116, 124] or *T. cutaneum* [125], as well as metabolic, genetic and evolutionary engineering [105, 126] strategies have been considered. To this end, some yeasts such as *C. oleaginosus* have been reported to grow on aromatic compounds from lignin degradation, potentially allowing full valorisation of lignocellulosic biomass [122, 123].

### Glycerol

**Fig. 4:**
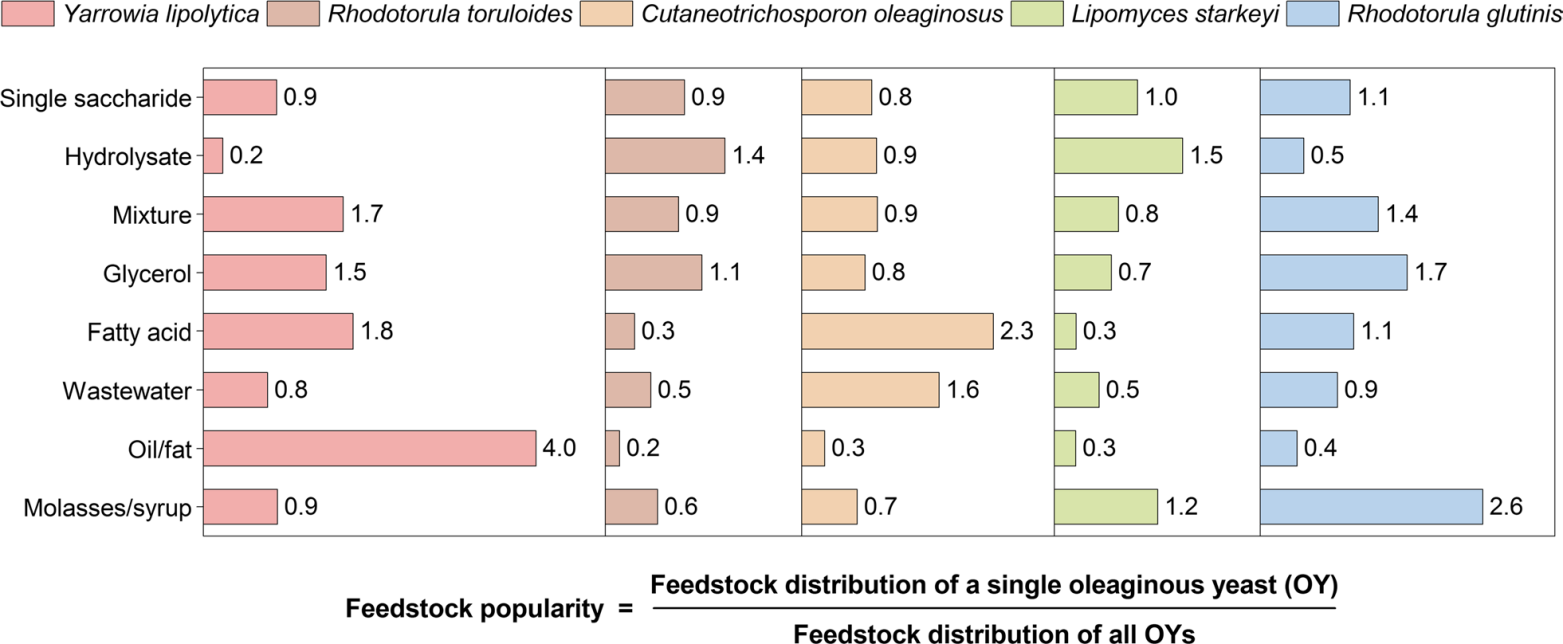
The feedstock preference of the most prominent oleaginous yeasts. Displayed is the feedstock popularity according to the depicted definition. As an example, *Yarrowia lipolytica* has been cultured on an oil/fat four times more often than the average yeast. The feedstock popularity indicates certain feedstock preferences of the specified yeast. For instance, when a fatty acid is the preferred feedstock, *Yarrowia lipolytica* and *Cutaneotrichosporon oleaginosus* are likely suitable yeasts. The feedstock distribution of all oleaginous yeasts can be reviewed in Fig. 3. Please see the corresponding caption for details of the carbon sources comprising each category. The full methodology used to collect and analyse the presented data is given in Additional file 1

Crude glycerol is considered a viable feedstock for microbial conversion, as it is often considered a waste stream due to various impurities from the major production processes [127–129]. Worldwide availability is over 2 million tonnes per year [127] with an average price of approximately US$ 220 per tonne (80% crude glycerol, 2018), though the price fluctuates widely depending on source and purity. Of the oleaginous yeasts cultured on glycerol, approximately half have been cultured on crude glycerol. Arguably, oleaginous microorganisms are ideal for the valorisation of industrial glycerol, as glycerol forms the structural backbone in TAGs, and is mainly sourced from biodiesel production, the most proposed application for yeast lipids. A microbial lipid process could therefore be integrated into a biodiesel production facility with the microbial lipids transesterified into excess biodiesel [129–131]. However, certain impurities such as methanol may inhibit the yeast requiring detoxification before fermentation [128, 129, 132]. *Rhodotorula* species and *Y. lipolytica* have been described to utilise glycerol very efficiently [90, 128, 133], and are therefore popular amongst researchers in this field using glycerol (Fig. 4). Some of the former, for instance, have been shown to produce increased amounts of conjugated linoleic acid (CLA) [90] and attain lipid yields around 0.20 g g^−1^ on crude [129, 134] and 0.27 g g^−1^ on pure glycerol [88].

### Oil/fat or fatty acids

Industrial and domestic waste fats, oil or grease can be converted by some oleaginous yeasts through the ex novo pathway [68, 135, 136]. However, in the EU around 90% of the collected used cooking oil (UCO) is already being used for biodiesel, wherefore the UCO price is beginning to track that of biodiesel (£400 to £700 per tonne in UK, 2014 [137]).

Volatile fatty acids (VFAs), on the other hand, are a very attractive substrate because they can be produced by a mixed microbial culture. The latter can convert an enormous range of substrates due to its diversity. Consequently, oleaginous yeasts have been grown on purified single VFAs such as acetate [47, 138, 139] or mixed VFAs [140] to determine suitability for cultivation on lignocellulosic hydrolysate or anaerobic digestion effluent. The cost of purified mixed VFA derived from macroalgae has been estimated at US$ 384 per tonne (2014) [141], but oleaginous yeasts could be cultured directly on anaerobic digestion effluent [139, 142]. A majority of studies on the conversion of fats and fatty acids have been concerned with *Y. lipolytica* [68] and *C. oleaginosus* [47, 138, 139] (Fig. 4), though some other yeasts, such as *Cryptococcus albidus,* have been cultured on VFAs [140].

### Whey or whey permeate

Whey and whey permeate are a waste product and one of the earlier feedstocks considered for oleaginous yeasts [18, 143]. Worldwide production is estimated around 190 million tonnes per year [144]. Although whey has such a huge potential as substrate, less than one percent of yeast lipid investigations have used this feedstock (Fig. 3). Microbial conversion requires a lactose-positive microbe that can also assimilate peptides and proteins. Whilst not common [145], oleaginous yeast that can metabolise lactose include *C. oleaginosus* [99, 143], *Kluyveromyces marxianus* [146] and *Y. lipolytica* [147].

## Oleaginous yeast species

**Fig. 5:**
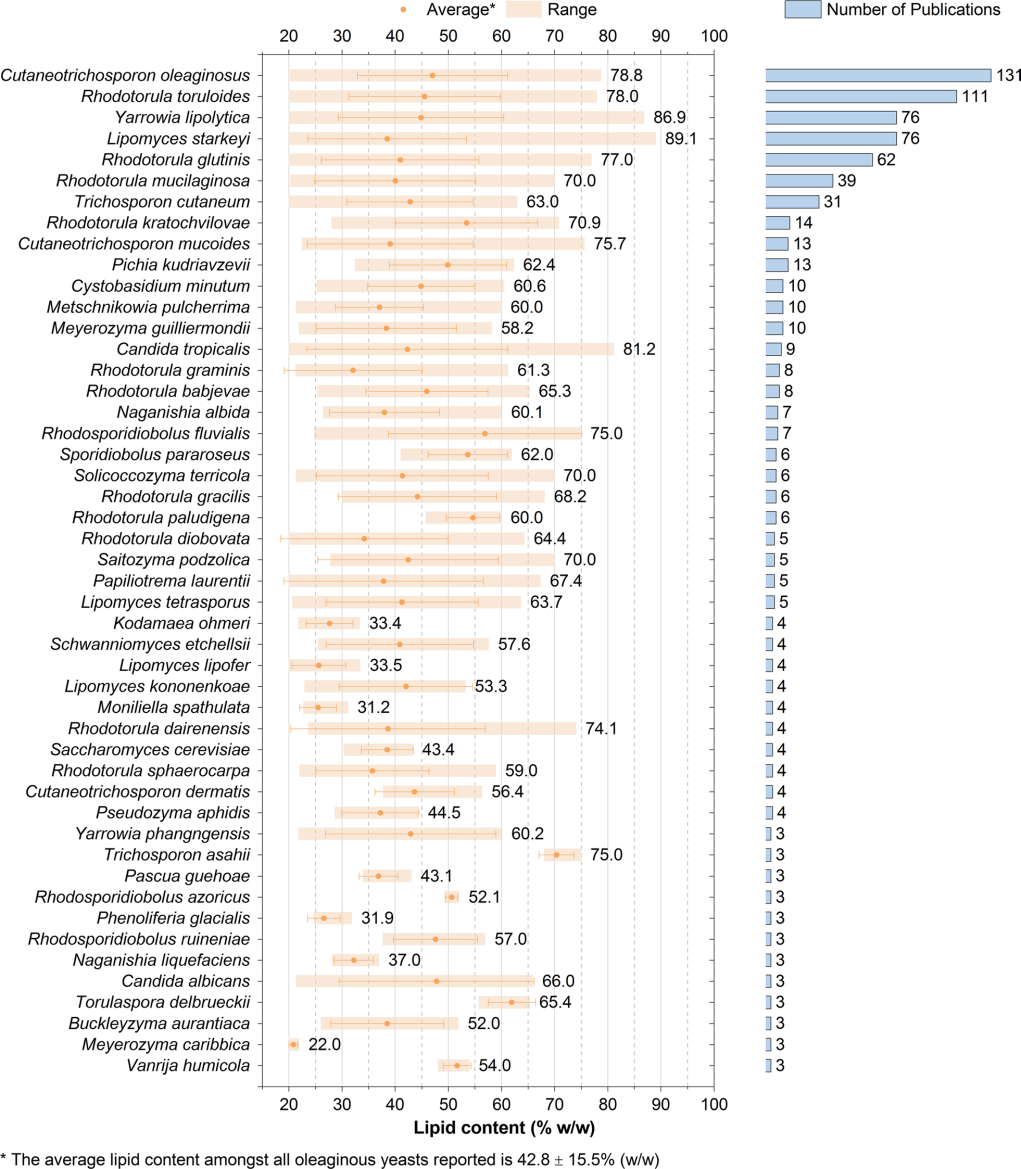
The confirmed native oleaginous yeasts. Included are all native yeasts or yeast-like species with a lipid content over or equal to 20% (w/w) reported in at least three publications and clearly identified with their generic name and specific epithet, as their current name according to the corresponding culture collection and MycoBank (48 yeasts in total). The range of the highest reported lipid contents including average and standard deviation, as well as the number of publications, where a strain of the species has been reported oleaginous, are depicted. For some species, such as *Saccharomyces cerevisiae*, not typically classified oleaginous, only certain strains have been shown to accumulate over 20% (w/w) lipid. The labels indicate the maximum lipid content recorded or the number of publications, respectively. Further identified oleaginous yeasts (less than three publications, 113 yeasts) are given in Additional file 1: Tables S3 and S4. The full methodology used to collect and analyse the presented data is given in Additional file 1

To date, over 160 yeast species have been reported in the scientific literature with lipid contents greater than or equal to 20% (w/w) (Fig. 5, Additional file 1: Tables S2, S3), making them oleaginous by definition [3]. Whilst some species can accumulate lipids over 70% (w/w) of their dry cell weight, the average has been 42.8% (w/w) ± 15.5% (w/w) (Fig. 5). Their lipid content, concentration and composition depend on several factors including yeast species, medium composition, and operational conditions. The variation in between species [148], but often even in between strains [149], is vast (Fig. 5, Table 1). To enhance industrial attractiveness and economic viability, robust oleaginous yeasts are required, which can grow to high cell densities, at a low pH and broad temperature range, on several carbon sources and under compromised sterility, as well as being genetically accessible [49, 112, 150, 151]. Oleaginous yeast species of major scientific interest include *Y. lipolytica*, *R. toruloides*, *C. oleaginosus*, *L. starkeyi*, and *R. glutinis*, together accounting for over 50% of oleaginous yeasts cultured, typically because of their high attainable lipid content, substrate suitability, growth performance, or genetic tractability (Table 1). Additionally, the first reported oleaginous yeast, *M. pulcherrima*, has recently re-attracted much attention due to the wide carbon assimilability [150, 152, 153], high inhibitor tolerance [54, 116], not strict dependence on nutrient limitation (e.g. C/N ratio) for oil accumulation [43, 104], and ability to naturally supress bacterial contamination [154–156] (Additional file 1: Fig. S1).

**Table 1:** The most prominent and promising oleaginous yeast species and their key characteristics

Yeast species	Popularity (% of oleaginous yeasts cultured)^a^	Max. lipid productivity (g L^−1^ h^−1^)^b^	Max. scale (L)^c^	Exemplary fatty acid composition (% w/w)					Major strengths	Weaknesses	Refs.
				16:0	16:1	18:0	18:1	18:2			
*Yarrowia lipolytica*	13.2 (7.9 + 5.3)	1.20 (GE)	5000	10.6	8.9	10.1	63.4	5.2	Genetic tractability; affinity towards hydrophobic substrate; co-product: citric acid	Limited substrate spectrum	[10, 41, 107, 164, 167]
*Rhodotorula toruloides*	13.0 (10.9 + 2.1)	1.48	1000	20.0	0.6	14.6	46.9	13.1	High yields; valuable co-product: carotenoids	Low inhibitor tolerance	[118, 177, 182, 256]
*Cutaneo-trichosporon oleaginosus*	11.6 (11.0 + 0.6)	1.00	250,000	18.9	0.2	24.8	45.7	4.6	Wide substrate spectrum (incl. lactose); high inhibitor tolerance; fast growth	–	[28, 30, 31, 118, 121]
*Lipomyces starkeyi*	7.8 (7.5 + 0.3)	1.60	21 (est.)	33.3	2.8	4.8	52.0	3.6	Co-fermentation of substrates	–	[109, 148, 198, 266, 316]
*Rhodotorula glutinis*	5.4 (5.1 + 0.3)	0.86	210 (est.)	15.4	7.2	9.1	63.5	1.8	Bio-control ability; valuable co-product: carotenoids	–	[97, 124, 204, 205, 290]
*Rhodotorula mucilaginosa*	4.1 (3.7 + 0.4)	0.33	12	19.6	0.8	6.1	41.8	27.0	Co-products: xylitol, carotenoids	–	[304, 312, 317]
*Trichosporon cutaneum*	2.8 (2.6 + 0.2)	0.17	14	30.0	–	13.0	46.0	11.0	Wide substrate spectrum (incl. lactose); high inhibitor tolerance	Dimorphism	[143, 318]
*Metschnikowia pulcherrima*	1.4 (1.2 + 0.2)	0.35 (EV)	500	38.5	2.5	4.6	47.6	–	Bio-control ability; valuable co-product: 2-phenylethanol; lipid accumulation on nutrient-rich feedstock; high inhibitor tolerance	Slow growth	[43, 104, 112, 124, 150, 156]

The fatty acid composition of the lipids includes palmitic (16:0), palmitoleic (16:1), stearic (18:0), oleic (18:1), linoleic (18:2) and alpha/gamma-linolenic acid (18:3)

^a^Split into native and genetically modified (genetically engineered, evolved, mutated) strains

^b^GE, genetically engineered; EV, evolved; please see Additional file 1: Table S5 for the average lipid productivity of the depicted species

^c^Working volume; est., estimated as 70% w/w of reactor volume

### Prominent and promising oleaginous yeasts

#### Yarrowia lipolytica

*Yarrowia lipolytica*, previously classified as *Candida lipolytica*, *Saccharomycopsis lipolytica* and *Endomycopsis lipolytica*, can be found in a wide variety of environments [26]. It is commonly isolated from fat- or protein-rich substances such as cheese, rather than sugar-rich material [157]. Its affinity towards hydrophobic substrates accelerated its industrial relevance to produce single cell protein (SCP) and citric acid from the late 1950s, as relatively cheap n-alkanes could be used as substrate [26, 157]. *Y. lipolytica* can produce significant quantities of SCO from other lipids through the *ex novo* conversion pathway [158]. However, despite the wide use of this species in the academic literature, only a few wild-type strains can accumulate above 20% (w/w) lipid through the de novo pathway when cultured on glucose or similar carbon sources [158–160]. Under nitrogen-limited conditions, *Y. lipolytica* typically accumulates lipids early in the growth cycle, before converting these compounds into a range of low-molecular weight compounds, such as citric acid [159, 161]. While some exceptions have been reported, generally de novo production of lipids with *Y. lipolytica* does not compare favourably with the other key strains discussed in the literature. However, its genetic accessibility makes *Y. lipolytica* an industrially-relevant versatile microbe that can produce a variety of valuable metabolites not limited to SCP and citric acid, but also carotenoids, erythritol, lipids, lipases, mannitol, and more [26, 162]. The native strain most frequently used for lipid production has been the French W29 (ATTCC 20460, American Type Culture Collection), but often less well-known strains or own isolates are used (Additional file 1: Fig. S5).

While its affinity towards hydrophobic substrate has still been used in recent yeast lipid studies [68], *Y. lipolytica* is also frequently cultured on glycerol for lipid production [163–165] (Fig. 4). It is rarely cultivated on hydrolysates, largely due to a limited xylose pathway [107, 166, 167] and inability to ferment cellulose [168]. Moreover, wild-type *Y. lipolytica* is known to be incapable of assimilating several monosaccharides including galactose, as well as di- and polysaccharides including sucrose, starch and inulin [169]. On culturing on glucose or similar carbon substrates, the produced lipids generally contain lower quantities of cellular oleic acid, with higher quantities of linoleic and alpha-linolenic acids detected [158, 170] (compared to those given in Table 1). Extremely high cell densities (194 g L^−1^) [171] and lipid productivities (1.2 g L^−1^ h^−1^) [41] have been achieved with GM *Y. lipolytica* (Additional file 1: Table S4). Due to its genetic accessibility, nearly 70% of the genetically engineered oleaginous yeasts cultured are of the species *Y. lipolytica*, with most originating in strain W29 (Additional file 1: Fig. S6). For the same reason, this yeast is not only considered for the production of commodity oil substitutes [41, 172], but also fatty alcohols [173] and unusual fatty acids such as long-chain PUFAs [162]. Several native genes may be overexpressed for increased lipid production [174]. Unsurprisingly, *Y. lipolytica* featured in the first-ever commercial oleaginous yeast process [9, 10]. Recent work has also demonstrated successful adaptive evolution strategies, increasing SCO production at the expense of citric acid biosynthesis [175].

#### Rhodotorula toruloides

Discovered oleaginous in 1944, *Rhodotorula toruloides*, also known as *Rhodosporidium toruloides*, *Rhodotorula rubescens* or *Rhodotorula gracilis* [16, 176], is well known for its industrial potential as a lipid producer achieving high lipid yields [88, 177], but also for the production of carotenoids and enzymes [178, 179]. Its distinctive red appearance, typical for *Rhodotorula* strains, is caused by considerable production of carotenoids (0.12 mg g^−1^ dry cell mass) [179]. The native *R. toruloides* strains most used for lipid production are DSM 4444 (German Collection of Microorganisms and Cell Cultures) and AS 2.1389 (China General Microbiological Culture Collection Center), together accounting for over 25% of native strains used (Additional file 1: Fig. S5).

With the lipid accumulation strongly carbon source-dependent, native strains are generally most suitable to single monosaccharides and polyols (Fig. 4). On glycerol, for instance, lipid yields of up to 0.27 g g^−1^ glycerol have been reported [88] and suitability to crude glycerol demonstrated [128, 130]. This is opposed to lignocellulosic hydrolysates: despite performing well on glucose and xylose separately, lipid accumulation is diminished when providing only arabinose or a mixture of these three sugars [180], and—strain dependent—further challenged through the presence of fermentation inhibitors [118, 181]. With wild-type strains some of the highest cell densities (185.0 g L^−1^) [53] and lipid productivities (1.48 g L^−1^ h^−1^) [182] to date have been achieved in fed-batch operation with oxygen-enriched air (Additional file 1: Table S4) [53]. The importance of *R. toruloides* for industrial biotechnology has been further increased as genetic tools have been developed [183–186]. For enhancing the inhibitor tolerance, for instance, mutagenesis, adaptive laboratory evolution and metabolic engineering have been applied [105, 187, 188]. In this respect, the evolved strain Y4 (from AS 2.1389) [177] is particularly popular amongst yeast lipid researchers (Additional file 1: Fig. S6).

#### Cutaneotrichosporon oleaginosus

Also known as *Cryptococcus curvatus*, but recently reclassified as *Cutaneotrichosporon oleaginosus* [189], this oleaginous yeast has been researched for decades. It was originally isolated from floors or drains of a dairy farm in 1978, and suggested as a suitable organism for producing either SCP or SCO from whey or whey permeate [143]. Deposited as *Candida curvata* D (ATCC 20509, American Type Culture Collection), it has since been known as *Apiotrichum curvatum*, *Cryptococcus curvatus*, *Trichosporon cutaneum*, or *Trichosporon oleaginosus* [190]. This original is still the main strain used, accounting for over 70% of native *C. oleaginosus* cultured (Additional file 1: Fig. S5).

*Cutaneotrichosporon oleaginosus’* major advantages are that it can grow relatively fast (lipid productivity of 1.0 g L^−1^ h^−1^ [28]), on a wide range of carbon sources [191] and possesses good inhibitor tolerance [118, 121, 122]. Indeed, for *C. oleaginosus* on average the highest lipid productivities have been reported (0.16 ± 0.17 g L^−1^ h^−1^, Additional file 1: Table S5). Due to its growth on xylose [89, 96], oligosaccharides [115], fatty acids [139, 142], as well as lactose [31, 89], commonly used substrates for *C. oleaginosus* are hydrolysates [192, 193], VFAs [47, 138], and comparably common, whey or whey permeate [28, 143] (Fig. 4). *Cutaneotrichosporon oleaginosus* has been shown capable of co-utilising various carbon sources, including glucose combined with acetate [192], glycerol [193] or xylose [96]. Acetate particularly is a fascinating feedstock for this yeast facilitating lipid accumulation [192] also under nutrient-rich conditions [81, 138, 171], as well as promoting extracellular lipid secretion [47]. This yeast has been grown in fed-batch culture up to a cell density of 104.1 g L^−1^ yet containing a remarkable lipid content of 82.7% (w/w) [40]; and featured in the scale-up (250 m^3^) when almost successfully attempting to produce a CBE [30]. Recently, *C. oleaginosus* has been subjected to genetic manipulation, which allows the targeted alteration of the fatty acid profile [194].

#### Lipomyces starkeyi

*Lipomyces starkeyi* is one of the few prominent oleaginous yeasts which have kept their name since discovery in 1946 [18]. Of the family Lipomycetaceae, which are strong lipid producers, *L. starkeyi* is regarded as the species with highest “biotechnological value” with highest attainable lipid content and inhibitor tolerance [110, 148]. The most used strains of this species are AS 2.1560 (China General Microbiological Culture Collection Center), DSM 70296 (German Collection of Microorganisms and Cell Cultures) and CBS 1807 (Central Bureau of Fungal Cultures) (Additional file 1: Fig. S5).

Amongst the yeasts discussed, *L. starkeyi* is known to often have relatively low growth rates. The species has been arguably the most used for the conversion of lignocellulosic hydrolysates (Fig. 4). Strains of *L. starkeyi* have been shown to simultaneously ferment xylose in combination with glucose [110], cellobiose [109] or acetate [119], but not arabinose [111]. Remarkably, co-utilisation of carbon sources is rare amongst other oleaginous yeasts [195]. This ability makes *L. starkeyi* very interesting for the continuous processing using mixed carbon sources such as lignocellulosic hydrolysates [196]. However, though capable of degrading common lignocellulosic hydrolysate inhibitors [110, 118], high concentrations potentially need to be addressed through detoxification [111], dilution [197], processing at a high pH [119] or high cell densities [54]. To this end, cell densities of 104.6 g L^−1^ have been achieved in a two-stage process with a combined lipid productivity of 0.79 g L^−1^ h^−1^ [198]. Transformation protocols have been published [199, 200], and used to have *L. starkeyi* producing increased amounts of long-chain PUFAs [201] and fatty alcohols [173].

#### Rhodotorula glutinis

Yet another species of the genus *Rhodotorula*, *R. glutinis* is a strong lipid, carotenoid and enzyme producer [202]. Discovered oleaginous in 1943 [15], it has previously been known as *Rhodotorula terrea*, *Torula glutinis*, *Saccharomyces glutinis* or *Cryptococcus glutinis*. Several strong lipid producing strains previously classified as *R. glutinis* have recently been reassigned as *R. toruloides* (Additional file 1: Table S4), such as ATCC 204091 (American Type Culture Collection, USA), CECT 1137 (Spanish Type Culture Collection, Spain) and NRRL Y-1091 (Agricultural Research Service, USA), or as *Rhodotorula mucilaginosa*, such as BCRC 22360 (Bioresource Collection and Research Center, Taiwan). Perceptually, this species therefore appears more popular amongst yeast lipid researchers than it is (Table 1) [203]. Due to its antagonistic traits, *R. glutinis* is frequently considered as biocontrol agent [204], hence for the non-sterile cultivation towards lipid production [97].

Although lipid yields of up to 0.19 g g^−1^ crude glycerol have been reported [134] and glycerol being frequently used by *R. glutinis* researchers (Fig. 4), poor utilisation has been demonstrated by some strains, potentially due to passive diffusion [205]. Moreover, molasses and syrups have been a comparably well used feedstock with this yeast (Fig. 4), with sucrose determined as a suitable carbon source [205, 206]. On this feedstock, a cell density of 106.0 g L^−1^ and lipid productivity of 0.86 g L^−1^ h^−1^ have been achieved [205]. Genetic tools are limited but under development for the *Rhodotorula* genera [207].

#### Metschnikowia pulcherrima

*Metschnikowia pulcherrima* is primarily isolated from fruit, flowers and in nectars occurring in a vast range of different strains [104]. The yeast underwent several re-classifications, previously termed *Candida pulcherrima*, *Torula pulcherrima*, *Torulopsis pulcherrima*, *Rhodotorula pulcherrima*, *Saccharomyces pulcherrimus* and *Cryptococcus castellanii*. It has an ability to outcompete other microbes through secretion of antimicrobial agents and iron sequestration [112, 154–156], hence its use in wine making as biocontrol agent [155]. Its osmophilia (growth below a water activity of 0.88 [54]) and acidophilia (growth below pH 2 [104]) further aid its effectiveness against microbial contamination. In addition to lipids, it produces a range co-products, most prominently high-value fragrance compound 2-phenylethanol (2-PE, up to 1.0 g L^−1^ [208]) and pulcherrimin [156]. Although its ability to form large lipid bodies was observed over 120 years ago [6, 209], it was not until recently that it was termed oleaginous [112, 152].

*Metschnikowia pulcherrima* grows on a range of carbon sources including mono-, di- and oligosaccharides, glycerol, lignocellulosic hydrolysate and wastewaters [104, 112, 116, 117, 152, 153]. As not necessarily requiring nutrient depletion for lipid accumulation [43], nutrient-rich feedstock is also suitable for lipid production [104, 116]. This yeast features high tolerance to inhibitors such as acetate, furfural or hydroxymethylfurfural [54, 116]. Compared to the other prominent oleaginous yeasts, *M. pulcherrima* features lower lipid yields (0.17 g g^−1^ glucose) largely due to polyol secretion [43, 54] and productivities (0.29 g L^−1^ h^−1^) [43], but the amongst the highest cell densities (122.6 g L^−1^) [54] of non-engineered oleaginous yeasts have been achieved with this yeast (Additional file 1: Table S4). Genetic tools have been limited to adaptive laboratory evolution [126], but genetic engineering techniques are under development [54].

### Discovery of new oleaginous species

Of the approximate 1500 known yeasts [210], almost 11% have been identified oleaginous so far (Fig. 5, Additional file 1: Tables S2, S3). They can be discovered in a range of different locations, with the majority isolated from plant matter or soil [211–213], but also in more extreme environments such as Antarctica [214] and the marine environment [215–217]. With the significantly increased research interest in this field (Fig. 1), the number of yeasts identified capable of producing over 20% (w/w) lipid will likely reach the 200 s in the short term.

Initially, oleaginous yeasts were simply discovered microscopically as forming a visible lipid droplet [6]. Nowadays, yeasts are typically grown on a nitrogen-limited medium, then stained for instance with Nile Red [218] or Sudan Black B [219, 220] to visualise the lipid droplet and examined microscopically. In random screening experiments, sometimes several hundred yeasts are isolated and then tested for their lipid production ability [212, 221]. Although this technique probably has a higher potential to deliver fascinating organisms which have never been thought to be oleaginous, typically only around 5.0 ± 3.1%^1^ of the screened organisms are found oleaginous [108, 136, 149, 213, 222, 223]. Targeted screening can dramatically increase the success rate. For instance, by selectively targeting yeast phylogenetically similar to, or in taxonomic order with known oleaginous species, up to 94% of isolated/acquired yeasts have been found to be oleaginous [107, 148, 211, 224]. In order to (further) simplify and accelerate the screening techniques, new technologies such as the screening on agar plates [221] are being developed.

With the sheer volume of yeast screened for oleaginicity in the past 143 years, it seems questionable whether a new species can be discovered with significantly superior characteristics for economic lipid production compared to the previously discussed prominent species. However, in light of favourable economics when producing high-value lipids such as long-chain PUFAs [217, 225] and/or valuable secondary metabolites [50, 226], diverted yeast species with such characteristics, such as *Wickerhamomyces siamensis* producing medium-chain TAGs [217]*,* could be of interest in this field. Therefore, in the discovery of new species, it is key for researchers to investigate the fatty acid profile and secondary metabolite production, placing the novelty of a potential production process over the simple assessment that the yeast is oleaginous.

## Influencing yeast lipid production

### Process development with oleaginous yeasts

Process parameters such as substrate, temperature, pH and dissolved oxygen (DO) can have an enormous effect on yeast lipid production parameters, including the fatty acid profile [24, 31, 43, 79, 227].

The limitation of key nutrients as a processing strategy to achieve a high lipid content through the de novo pathway has been known for over a century [6, 11] and is applied to the vast majority of yeast lipid processes [224]. Productivities can be enhanced when cultivating on nutrient-rich media [43, 171], but only a few oleaginous yeasts are known to accumulate lipids under those conditions on sugar [43–45], and some also on acetate [81, 138, 171]. A switch or combination of the carbon source is often employed to increase yields or obtain a different fatty acid profile [56, 69, 87–90]. The feedstock range for oleaginous yeast is vast (Fig. 3), but depends on the capabilities of the specific yeast [151]. Huang et al*.* [47] demonstrated that the extracellular secretion of lipids can be facilitated through culturing on acetate with strains of certain species such as *C. oleaginosus*. The use of a hydrophobic substrate via the ex novo pathway may be useful in altering the fatty acid profile of waste oils or fats [68, 69]. To alter the fatty acid profile of the lipid, also enzyme inhibitors such as specific fatty acids can be added to the broth [72, 228]. This strategy was considered when attempting to increase the stearic acid (C18:0) content for the production of a CBE with *C. oleaginosus* [229], but they increase the process costs [33] and some of them are toxic.

Most oleaginous yeasts are mesophilic and cultured around 30 °C [43, 230], but there are records of oleaginous yeasts being cultured as low as − 3 °C (psychrophilic *Rhodotorula glacialis*) [231] and as high as 45 °C (*Blastobotrys raffinosifermentans*, *Blastobotrys adeninivorans*) [232]. Typically slightly acidic conditions (pH 5 ± 2) are applied [43, 203, 227], but oleaginous yeasts have been cultured in the range of pH 1.9 (*M. pulcherrima*) [104] to 12 (*R. toruloides*) [233]. In several cases, a high DO (over 30%) has been reported to facilitate lipid accumulation [31, 104, 234], but with *Y. lipolytica*, for instance, lipid yields can be increased through regulating the DO and thereby minimising citrate formation [172]. Finally, the extent of lipid accumulation, which can be altered through the discussed process parameters, forms a strong correlation with the fatty acid profile for some yeasts [235].

**Fig. 6:**
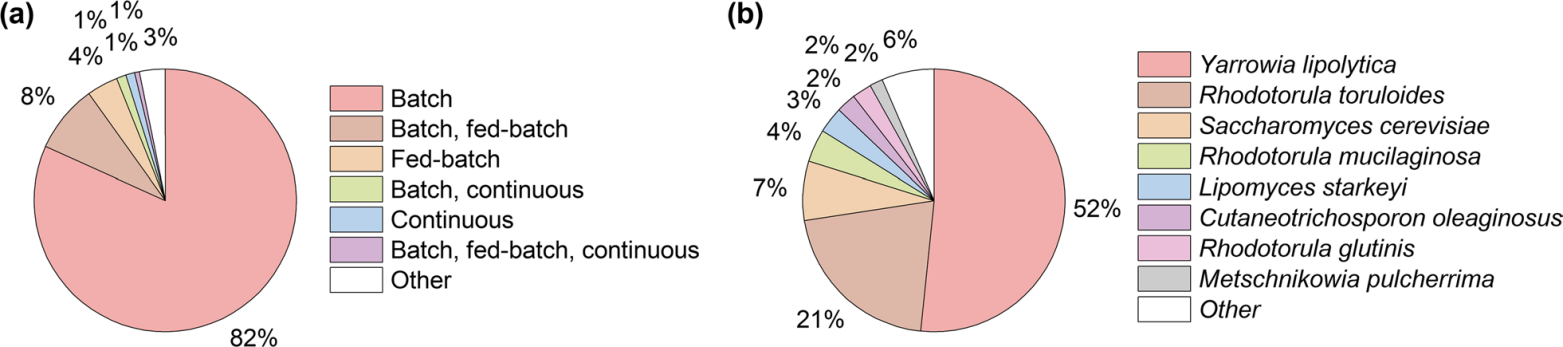
Influencing yeast lipid production through process development and genetic modification. **a** Percentage of oleaginous yeasts cultured in different operation modes used in research and **b** percentage of oleaginous yeast species used for genetic modification (including genetic engineering, evolution, and mutation). The full methodology used to collect and analyse the presented data is given in Additional file 1

Lipid production parameters can also be influenced by the operation mode, which has been extensively compared by Abeln and Chuck [54] and Anschau et al*.* [196], for example through facilitating high cell densities (Additional file 1: Table S4). For instance, in fed-batch and continuous operation generally higher lipid productivities have been achieved compared to batch processing (Additional file 1: Fig. S7). Most oleaginous yeasts have been fermented solely in batch (over 80%), followed by batch and fed-batch, and only fed-batch operation (Fig. 6). Around one percent have been cultured in batch and continuous operation as well as only continuous, with other (combinations of) modes rarely used. Flasks have been the largest used vessel for around two-thirds of oleaginous yeasts reported, and in those, an astonishing 97% of oleaginous yeasts cultured solely in batch mode (Additional file 1: Fig. S8). Although batch fermentations have increased simplicity and flexibility, and reduced risk of contamination, higher productivities and cell densities are prevented by the high osmotic pressure of concentrated substrates [54, 177]. In fed-batch mode, often higher lipid production rates can be achieved due to the increased number of cells synthesising lipids [53, 71]. Typically, nutrient-rich conditions are supplied initially to promote cell proliferation, after which only the carbon source is fed [71]—set to achieve extreme cell densities of up to 185.0 g L^−1^ [53] and lipid productivities up to 1.2 g L^−1^ h^−1^ [41]. When cultured in stirred tank reactors (24% of oleaginous yeasts), 40% of yeasts are cultured involving fed-batch cultivation (Additional file 1: Fig. S8). Continuous fermentations have been performed to learn more about the mechanisms of yeast lipid synthesis or a specific organism, or increase the productivity [24, 54, 99, 138, 236, 237]. With *C. oleaginosus*, for instance, Ykema et al*.* [28] achieved lipid productivities of up to 1.0 g L^−1^ h^−1^ [28], and with *M. pulcherrima* the lipid production rate could be doubled compared to the batch (0.37 g L^−1^ h^−1^) [54]—in both cases in flow fermentation with cell retention. For oleaginous yeasts requiring nutrient limitation, continuous cultivation typically requires adapted strategies for advanced lipid production as either growth and lipid production need to be separated in two vessels [236] or the process run at lower dilution rates and low nutrient loadings [99, 237, 238]. To this end, oleaginous yeasts able to produce lipids under variable nutrient loadings [43–45, 117], or applying a “carbon-restrained mode” using acetate as feedstock [239] could be beneficial.

To aid further process development, a number of studies have recently developed kinetic models for lipid production. These studies demonstrate the link between the lipid accumulation kinetics and various processing parameters demonstrating key potential bioprocess improvements and presenting predictive modelling scale-up tools [240–242].

### Modification of oleaginous yeasts

Around 7% of oleaginous yeast publications have been concerned with genetic modification, mainly through genetic engineering (nearly 70% of genetically modified oleaginous yeasts), but also evolution or mutation—typically to increase the lipid content and productivity, modify the lipid composition, or increase the inhibitor tolerance (Additional file 1: Fig. S6). For evolution or mutagenesis, for instance, Liu et al*.* [243] developed an interesting strategy involving the targeting of lipid-rich cells ‘floating’ due to buoyancy. The most frequently genetically modified yeast is *Y. lipolytica* (over 50% of modified yeasts) due to advanced development of genetic tools, though significant interest in *R. toruloides, L. starkeyi* and more recently *M. pulcherrima* is developing [156, 244] (Fig. 6). Genetic engineering tools have been employed harnessing the knowledge of the fatty acid pathways (see section “Fatty acid synthesis in oleaginous yeast”).

To enhance lipid production of native fatty acids (typically C16:0 to C18:3), often existing genes encoding for enzymes involved in the fatty acid pathway are overexpressed or deleted [174]. For example, with *Y. lipolytica*, a lipid content of 77% (w/w) has been achieved de novo through overexpression of heterologous diacylglycerol acyltransferase (DGA) from *R. toruloides* (DGA1) and *Claviceps purpurea* (DGA2), and deletion of the native TGL3 lipase regulator. In a fed-batch fermentation the strain achieved a lipid concentration of 85 g L^−1^ and productivity of 0.73 g L^−1^ h^−1^ [55]. Simultaneous overexpression together with the delta-9 stearoyl-CoA desaturase and acetyl-CoA carboxylase genes led to a high lipid yield of 0.23 g g^−1^ [172]. Qiao et al*.* [41] have since increased the lipid productivity attainable to 1.2 g L^−1^ h^−1^ through incorporating pathways synthesising lipid precursors NADPH or acetyl-CoA. Commonly however, the endogenous cell metabolism in these engineered strains produces harmful metabolites that “compromise cell fitness and productivity” [42]. Unsaturated lipids are highly prone to these oxidative degradative pathways and the resulting products further damage the productivity. Xu et al*.* determined that coupling glutathione disulfide reductase and glucose-6-phosphate dehydrogenase with aldehyde dehydrogenase was an effective method to reduce the effect of the oxygen and aldehyde stress in *Y. lipolytica*. Using this engineered strain, the authors reported elevated lipid titers (72.7 g L^−1^), lipid content (81.4% w/w) and productivity (0.97 g L^−1^ h^−1^) in lab-scale bioreactors [42].

Excitingly, Ledesma-Amaro et al*.* [48] have achieved the secretion of lipids, particularly free fatty acids (FFAs), through disrupting the synthesis of acyl-CoA from FFAs and beta-oxidation. In bacteria, fatty acid synthesis is carried out by fatty acid synthase (FAS) type II, encoded by a different set of enzymes compared to yeast. The thioesterases are responsible for directly releasing the FFAs into the cytoplasm. In *E. coli* the overexpression of this family of genes have been achieved, demonstrating some, limited, FFA synthesis and excretion from the cell [245]. The authors therefore mimicked this system in *Y. lipolytica* almost completely removing the triglyceride production and by overexpression of endogenous, re-localised or heterologous acyl-CoA thioesterases, removed the ability to degrade the FFAs, produced strains capable of synthesising FFAs and extracting the lipid into the supernatant.

Additionally, Xu et al*.* [46] assessed a multitude of strategies to engineer alternative pathways in *Y. lipolytica.* Acyl-CoA/acyl-ACP (acyl carrier protein) processing enzymes, in the cytoplasm, peroxisome, or endoplasmic reticulum were used to produce alkyl esters and alkanes. Activation of endogenous FFAs and the reduction of fatty acyl-CoAs allowed the synthesis of fatty alcohols. The authors also manipulated the chain length of the lipids, through engineering a hybrid FAS. Finally the manipulation of cytosolic acetyl-CoA pathways partially decoupled lipogenesis from nitrogen starvation allowing a simpler lipid accumulation process [46].

To produce non-native fatty acids, exogenous genes are required and are typically obtained from plants or moulds [194, 201]. For example, erucic acid (C22:1) and nervonic acid (C24:1) are highly sought targets for many industries. These were produced in *R. toruloides* through ectopic integration and heterologous expression of fatty acid elongases, namely 3-ketoacyl-CoA synthases, from various plants. Encouragingly, oil titers achieved remained high, up to 50 g L^−1^, and contained up to 30% of the target fatty acids in the lipid portion [186].

Although bacterial lipids are unsuitable for human consumption, and triglyceride production in bacteria rare, certain genes might be useful to transfer into yeasts [246]. Algal genes are particularly useful for the production of PUFAs, which are non-native to most oleaginous yeasts [194]. In *Y. lipolytica* researchers at DuPont overexpressed several desaturase and elongase genes to generate a host suitable for commercial EPA production, producing EPA at over 50% (w/w) lipids [9, 10]. The resulting strain contained 41 copies of 19 different genes. Other examples include the expression of multiple plant fatty acid elongase genes in *R. toruloides* to produce long-chain MUFAs (C22:1 and C24:1) [186]; a flax delta-15 desaturase gene in *L. starkeyi* to obtain increased amounts of ALA, which in turn was converted into EPA and DHA [201]; a fungal delta-12/omega-3 desaturase and an algal delta-9 elongase gene in *C. oleaginosus* to enhance ALA production and produce non-native long-chain PUFAs (C20:2 and C20:3), respectively [194]. The production of CLA, produced by native *Rhodotorula* strains [90], has also been achieved through the expression of a bacterial linoleic acid (LA) isomerase gene, both in *Y. lipolytica* [247] and *C. oleaginosus* [194].

Due to their natural advantages particularly in feedstock selection and inhibitor tolerance, Spagnuolo et al*.* [248] argued that *C. oleaginosus*, *R. toruloides*, and *L. starkeyi* would be better hosts for lipid synthesis than engineered *Y. lipolytica* if advanced genetic engineering tools were available. With native *C. oleaginosus*, also similar lipid productivities to engineered *Y. lipolytica* have been achieved (1.0 g L^−1^ h^−1^ [28] versus 1.2 g L^−1^ h^−1^ [41], respectively). Indeed, *C. oleaginosus* often delivers the best lipid production parameters amongst wild-type yeasts [123], including the highest productivities (Additional file 1: Table S5).

The most widely used organism for biotechnology is *S. cerevisiae*, though is not classified as oleaginous, some strains can produce up to 20% lipid [123, 249]. Lipid production has been improved dramatically in *S. cerevisiae* through modifying the yeasts’ diacylglycerol acyltransferase. For example, Kamisaka et al*.* overexpressed ga1p lacking the N-terminal 29 amino acids (Dga1∆Np) in a *S. cerevisiae* mutant. The resulting strain had the ability to produce a lipid content of up to 45% (w/w). The authors further reported the deletion of the 3′ terminal region of the *dga1* ORF, and it was this, rather than abrogation of genomic Dga1p expression, that had the large effect on increasing the lipid accumulation [250]. Building on this work, researchers at NREL further increased the lipid productivity in *S. cerevisiae* by knocking out the ADP-activated serine/threonine kinase (*SNF1*) of a strain. This increased lipid accumulation from 20% (w/w) to 35% (w/w) of dry cell weight. This strain was also engineering to produce lipids from xylose as the only carbon source. The lipid content was further increased to 50% (w/w) with the overexpression of diacylglycerol acyltransferase (*DGA1*) genes [251].

## Yeast lipid applications

In the last 30 years world production of oils and fats has increased from around 83.5 to over 223.0 million tonnes [252] and is expected to increase substantially in the decades ahead [253]. Yeast lipids could be used to meet this increased demand for oil and partly substitute or complement more traditional sources of vegetable oils or fats.

Biodiesel is the most frequently proposed application followed by food/supplements (e.g. cocoa butter equivalent, palm oil substitute, PUFAs), oleochemicals and animal feed (Additional file 1: Fig. S9). Remarkably, over 75% of publications have named biofuel as one potential application for the yeast lipid (Additional file 1: Fig. S4).

### Biodiesel and hydroprocessed fatty acid esters

Current global production of biodiesel is around 44 billion litres [254]. Approximately 75% of biodiesel is derived from vegetable oils (first-generation), which totals around 14% of all vegetable oil [254]. Consequently, the second-generation biodiesel introduced non-edible plants, waste oils and animal fat as biodiesel precursors [255]. The EU approved a revised Renewable Energy Directive (RED II): whilst increasing the biofuel fraction in transport to 14% by 2030, it is set to limit food and feed crops as feedstock (7%), phase out high indirect land use change (ILUC) risk feedstock such as palm oil, and increase “advanced” feedstock such as algae and bio-wastes (3.5%) [37]. This can give another boost to the developing yeast lipid technology.

Microbially derived biodiesel is often referred to as a third-generation biofuel [8, 256]. Although promising, the major consensus seems to be that microbial biodiesel production can only become a reality with subsidies [38, 257] and if the price of first-generation biodiesel (US$ 990 per tonne, 2019 [254]), and ultimately petroleum diesel increases [7, 8]. An unrealistic lipid productivity for the yeast of 7.5 g L^−1^ h^−1^ and a sugar price of US$ 70 per tonne have been proposed to reach profitability (2013) [257]. As biodiesel does not need to be food-grade, the operation cost could potentially be reduced through semi-sterile operation when coupled with antagonistic oleaginous yeasts such as *M. pulcherrima* or *W. anomalus* [56, 112]. Finally, to reduce processing steps, direct transesterification of the lipid without extraction into fatty acid methyl/ethyl esters has been investigated [131, 258, 259].

### Cocoa butter equivalent

Extracted from cocoa beans, cocoa butter has a typical cocoa flavour and is solid at room temperature as it contains around 60% saturated fatty acids (Additional file 1: Table S1). A CBE must have a similar TAG structure (mostly saturated‐unsaturated‐saturated) to mimic the correct properties [73, 260]. Due to the relatively high price of cocoa butter, substitutes are frequently used, mostly derived from other plants such as palm [261]. This is a market opportunity for the relatively expensive yeast lipid, which can deliver higher quality CBEs [33]. Following the pioneering industrial work in the 1980s, further research has attempted to develop a CBE using *C. oleaginosus* [73]*, Saitozyma podzolica* [222] or *Y. lipolytica* [68, 87], with a GM version of which over 20% higher productivities have been achieved compared to the original process [41]. However, currently it is not clear whether a GMO oil will be publicly accepted for food purposes [262, 263] or a wild-type strain needs to be used. For example, in a joint venture TerraVia (acquired by Corbion in 2017) and Bunge (Chesterfield, USA) marketed an algae butter AlgaWise™ as a CBE, containing about 70% stearic-oleic-stearic TAGs. However, produced by GM heterotrophic microalgae from Brazilian sugarcane, commercial interest in this product has all but vanished.

### Palm oil substitute

Extracted from the mesocarp of the oil palms’ fruit, palm oil is also high in saturated fatty acids with around 44% palmitic acid, giving a semi-solid appearance around room temperature (Additional file 1: Table S1). Over 69 million tonnes are produced annually which accounts for approximately a third of all vegetable oils [252]. Palm oil is primarily used for food purposes, in personal care products and increasingly for biofuels [37, 38, 264]. Mainly due to the palms’ high productivity per hectare (around four tonnes oil per hectare [265]), it is also the cheapest vegetable oil. However, the steep increase in demand has led to substantial logging of tropical rainforest, primarily in Asia and South America [264].

Certain wild-type oleaginous yeasts such as *Lipomyces lipofer* [134], *L. starkeyi* [109, 134, 148, 196, 266], *Macalpinomyces spermophorus* [223], *M. pulcherrima* [54] and *R. glutinis* [120, 267] have been shown to produce oil similar in composition to palm oil. This oil has the potential to be used for further processing or as a cooking oil in an unrefined state. However, despite potentially possessing a higher value such as through being tailorable, organic and deforestation-free [268], commercialisation is currently elusive due to the low cost of palm oil [50]. The pressure to reduce production costs mean that cheap substrates [109, 134] and advanced processing technology are essential [112]. To meet sustainability objectives, industry demand for such a product already exists. For example, AlgaWise™ was also marketed as alternative to palm oil, with Unilever, Mitsui, AkzoNobel and Ecover as (potential) commercial consumers [269], and a host of new companies such as C16 Biosciences are now operating in this space.

### Polyunsaturated fatty acids

The long-chain PUFAs GLA (C18:3, omega-6), EPA (C20:5, omega-3), ARA (C20:4, omega-6) and DHA (C22:6, omega-3) are dietary essential under certain medical conditions, but also provide health benefits [270–272], especially in infants [273, 274]. They are often termed conditional essential [275]. As of now, EPA, ARA and DHA are not commercially available from higher plants [276, 277], but Nuseed (Laverton, Australia) is in the process of commercialising a DHA-enriched oil from GM canola named Nutriterra^®^ [278].

In native yeast, however, these long-chain PUFAs are not typically produced or only in small amounts [31, 59, 217, 233], wherefore microbial GLA, ARA and DHA are commercially produced by a range of algae and moulds with DSM (Heerlen, Netherlands) arguably being the major global player [59]. As for the remaining EPA it is understood that no natural organism has yet been identified as suitable industrial production host producing predominantly EPA [67]. Therefore, DuPont used the genetic engineering tools available for *Y. lipolytica* [9, 10]. Their commercial strain produced EPA at around 50% total lipids [225]. Microalgal EPA-rich oils which are currently marketed include AlmegaPL^®^ by Qualitas Health (Houston, USA) containing 25% EPA (no ARA, DHA) from *Nannochloropsis* sp. and life’s™ OMEGA by DSM containing increased DHA and EPA, as it appears produced by GM *Schizochytrium* sp. [279].

### Animal feed

Oleaginous yeasts may be used as animal feed or for aquaculture as a source of protein, vitamins, antioxidants and fatty acids [213]. Benefits of enriching animal feeds with long-chain PUFAs have also been demonstrated with a wide range of terrestrial livestock, including poultry, pigs, and dairy animals [280]. Consequently, the venture Verlasso^®^ was built upon using DuPont’s GM *Y. lipolytica* strain [225] to increase the amounts of EPA and DHA in the salmon’s diet. Similarly, microalgal omega-3 oils have been developed for animal feed and are still being produced, for example DHA Natur™ by ADM Animal Nutrition (Chicago, USA), AlgaPrime™ DHA by Corbion (Amsterdam, Netherlands), DHAgold™ by DSM Nutritional Products. The Evonik/DSM joint venture Veramaris (Delft, Netherlands) uses non-GMO *Schizochytrium* sp. for this purpose [278].

Oleaginous yeasts can deliver nutritional benefits in addition to their lipids [213], for example when containing a balanced range of amino acids (AAs), high amounts of limiting AAs, and/or antimicrobial compounds [117]. Oleaginous yeast protein typically has a high lysine content, with the methionine content also similar to soybean meal protein, underlining its potential for feeds supplementation [117, 281, 282]. Using the whole cell biomass preserves a higher nutritional value for many of the cell-contained nutraceuticals. Antimicrobial compounds produced by yeast may also benefit a feed mix in terms of preservation and could potentially lead to higher resistance towards diseases. Oleaginous yeasts with known antimicrobial properties are *M. pulcherrima* [154–156, 283] and *Wickerhamomyces anomalus* [56, 283, 284].

## Economic considerations

In general, oleaginous yeasts have advantages over heterotrophic algae, achieving higher cell densities and being less susceptible to contamination [8, 49], and also over moulds, achieving higher growth rates and being easier to handle at scale [77].

Estimates for the cost of manufacture range from US$ 1300 to 9900 per tonne oil, largely depending on which lipid production data the study was based on, the valuation of co-products such as spent biomass or other metabolites, the feedstock and fermentation system used, the production scale, as well as the approach to capital investment (Table 2). Recently, Karamerou et al*.* presented a techno-economic model comprising of the largest possible reactor configuration with a hypothetical heterotroph working at the biological limit for biomass yield and lipid production, on the cheapest possible sugar source. The authors noted that while clearly impossible to recreate in reality this would be the theoretically cheapest lipid price, given as $1200 per tonne [285] for an unrefined oil, produced without valorisation of the other components produced from the system. Therefore, oleaginous yeasts could potentially be used to produce high-value lipids, meaning those missing an abundant source [2]. To directly substitute a commodity oil such as palm or soybean oil, the estimated yeast lipid production costs are currently too high (Additional file 1: Fig. S9, Table 2).

**Table 2:** Recent estimates of yeast lipid production cost

Feedstock	Study year	Reactor type	Annual production (t oil/y)	Yeast lipid productivity (g L^−1^ h^−1^)	Valued co-products	Cost (US$/t oil)	Refs.
Zero-value feedstock	2008	–	–	–	–	3000 (COM)	[7]
Glucose (US$ 400/t)	2014	STR	10,000	0.54	–	5480 (COM)	[49]
Zero-value carbon source	2014	STR	10,000	0.54	–	3410 (COM)	[49]
Zero-value carbon source	2014	STR	10,000	2.50	–	1760 (COM)	[49]
Zero-value carbon source	2016	STR	10,260	0.21	Animal feed	2350 (BP)	[265]
Zero-value carbon source	2016	OP	10,260	0.21	Animal feed	1723 (BP)	[265]
Zero-value carbon source	2016	OP	7900	0.13	Animal feed	2290 (BP)	[265]
Molasses (US$ 100/t)	2016	STR	16,720	0.42	Animal feed	1300 (SP)	[98]
Sucrose (US$ 258/t)	2019	STR	10,000	0.52	Animal feed, 2-phenylethanol	5240 (COM)	[50]^a^
Sucrose (US$ 258/t)	2019	OP	7700	0.40	Animal feed, 2-phenylethanol	5690 (COM)	[50]^a^
Wheat straw (US$ 78/t)	2019	STR	10,000	0.52	Animal feed, 2-phenylethanol	5690 (COM)	[50]^a^
Distiller’s dried grains with solubles (US$ 255/t)	2019	STR	10,000	0.52	Animal feed, 2-phenylethanol	9870 (COM)	[50]^a^
Glucose (US$ 400/t)	2020	STR	40,000	0.54	–	4100 (MSP)	[52]
Glucose (US$ 400/t)	2020	STR	2000	0.54	–	5800 (MSP)	[52]
Glucose (US$ 100/t)	2020	STR	40,000	0.54	–	2500 (MSP)	[52]

*STR* stirred tank reactor, *OP* open pond, *COM* cost of manufacture, *BP* break-even price, *MSP* minimum selling price, *SP* selling price

^a^Prices converted with a €1 to US$ 1.12 exchange rate (2019 average)

### Feedstock cost

As heterotrophic microorganisms, yeasts require an organic carbon source, which can become one of the main operating costs of the process. In yeast lipid processes fermenting glucose, for example, Koutinas et al*.* estimated its cost to contribute to around 40% of the total lipid production cost [49]. Even with the best yielding yeasts, 4 to 5 tonnes of saccharides are required to produce one tonne of oil, with sugars from sugarcane or corn costing around US$ 430 per tonne (2013) [257].

Lignocellulosic biomass has been investigated largely in the last two decades [286] (Fig. 3) due to its low cost of around US$ 50 to 60 per tonne (2007/2008), excluding the cost of depolymerisation to release the saccharides [106, 287]. Researchers at the National Renewable Energy Laboratory (NREL; Golden, USA) have estimated the sugar selling price for a dilute sugar solution (127 g L^−1^) around US$ 255 per tonne (2007), and for a concentrated sugar solution (487 g L^−1^) US$ 315 per tonne [106]. Potentially further development including co-product valorisation may reduce the price to US$ 79 per tonne [287]. Industrial and agro-industrial wastes are a further possibility examples include crude glycerol [163], whey or whey permeate [144], sewage sludge [288], wastewaters [289, 290] and food waste [233]. Some waste streams may even occur at a negative cost, when they require additional treatment for safe disposal [49], though treatment may still be required after fermentation and they would be unsuitable for producing a food-grade oil [66].

Lower lipid yields, productivities and different lipid composition are common when cultured on waste substrates due to fermentation inhibitors, lower or higher (non-limiting) nutrient contents [92, 104, 108, 132]. The technical challenge here is to find a suitable host, native [43–45] or GM [46, 105], and conduct process development to bridge the gap [43, 151, 171]. *Trichosporon cutaneum* [125] and *M. pulcherrima* [54, 116, 124], for instance, have remarkable inhibitor tolerance, and the latter additionally accumulates lipids in nutrient-rich media [43, 104, 116].

### Capital and other operating cost

The type of bioreactor has the largest influence on the capital cost for the bioconversion [49, 98]. Capital cost of a single 250 m^3^ field-erected fermenter was estimated around US$ 3.6 million (2010) [49], accounting for up to 90% of the equipment cost in yeast lipid production [98]. Similarly, Koutinas et al*.* [49] determined that the capital cost of field-erected fermenters contribute immensely to the cost of biodiesel production via yeast lipids amounting to over 70% of the total capital cost or almost 90% when including the air compressors. These values are far higher when compared to anaerobic fermentation. Parsons et al*.* [291] argued that the capital cost currently “prohibit serious investment” in SCO technology.

Lower cost alternatives are needed for commercialisation of a yeast-derived commodity oil substitute. Such fermentation technology could include alternative reactor systems such as bubble columns [31] and airlifts [22]. For example, Braunwald et al*.* estimated that the break-even price for a microbial oil could be around 27% lower when the yeast was cultured in open pond systems compared to conventional STRs [265].

High productivities have been deemed necessary to increase the economic feasibility of yeast lipid production [49, 50, 292], but they are accompanied by increased oxygen requirements, necessitating high aeration, agitation and possibly even supply of oxygen-enriched air [43, 53, 237]. Consequently, operating and capital cost increase when achieving high productivities of yeast [293]. At a yeast biomass productivity of 0.8 g L^−1^ h^−1^, the electricity cost of the fermenters including air compressors has been estimated at 63% of the total utility cost towards biodiesel production [49]. Therefore, within the scope of technical feasibility and investment potential, a trade-off between productivity (oxygen input), operating and capital cost might be necessary.

**Fig. 7:**
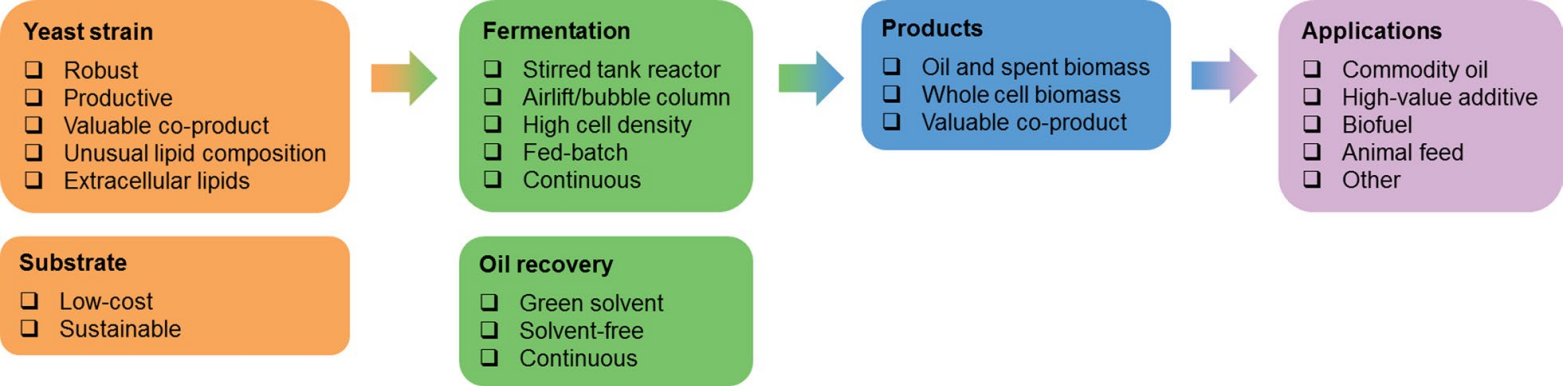
The proposed areas of future oleaginous yeast research towards producing a commodity oil substitute. Research in process or product development of oleaginous yeasts should ideally focus on the depicted areas

Finally, potentially more robust oleaginous yeasts are required to further reduce the capital and operating cost associated to maintaining sterility [49, 132] (Fig. 7). *Metschnikowia pulcherrima* or *W. anomalus* have been shown to produce substantial amounts of lipids on low-cost substrates [56, 112, 116, 294] and feature paramount biocontrol abilities [154, 283, 284]. For increased sterility, salt may be deployed as additional bacterial inhibiting agent, potentially promoting lipid production [128, 295]. In a design with (capital cost-heavy) field-erected fermenters, the complete omission of sterilisation could save 4.0% of capital and 1.2% of utility cost in yeast lipid production for heat exchange alone [49], with the capital cost further reduced through the vessel not requiring vacuumisation [293]. Potentially, idle times and production loss upon accidental non-sterile working would also be reduced. It should be emphasised however, that the complete omission of sterilisation is impractical from a safety point of view, unless effective biocontrol ability against any possible contamination was proven, and that for a food-grade process, strict regulations have to be met regarding contamination.

### Lipid yield and productivity

Theoretically, the highest attainable lipid yield in the form of TAGs is around 0.32 g g^−1^ from glucose, different from ethanol (0.54 g g^−1^), xylose (0.34 g g^−1^) and glycerol (0.30 g g^−1^), according to the stoichiometric calculation neglecting cell growth and maintenance [57, 77]. The highest lipid yields from glucose have been limited to around 85% of the theoretical (Additional file 1: Table S4) and generally are limited to approximately 0.20 g g^−1^ saccharide (Additional file 1: Fig. S10a). As some of the feedstock energy is required to produce biomass and for cellular process, lipids yields even nearer the theoretical may only be achieved when they are decoupled from biomass production, for instance through extracellular lipid production with continuous extraction [47, 48, 257]. Researchers at NREL determined that the yield should be around 0.28 g g^−1^ from lignocellulosic sugars targeting a US$ 5 per gallon gasoline equivalent [296]. Especially for a costly feedstock, a low lipid yield can have a detrimental effect on the economics [50].

The lipid content is often used to evaluate attractiveness of an oleaginous yeast, but the lipid productivity is the more pertinent metric due to its impact on the economics: higher productivities can moderate high capital cost over plant production time [49, 50, 292]. Most lipid productivities reported to date have been below 0.1 g L^−1^ h^−1^ (nearly 75%) and only 3% above 0.5 g L^−1^ h^−1^ (Additional file 1: Fig. S10b). To a large extent, this cluster at low productivities is due to non-optimised fermentation in shake flasks with lower productivities due to oxygen limitation and pH drift compared to STRs (Additional file 1: Fig. S7). Though it should be recognised that oleaginous yeasts have different natural productivities, genetic engineering [41, 172] and processing strategies such as high cell density fermentation (Additional file 1: Table S4) and providing nutrient-rich conditions [43, 171] have been proven as excellent measures to increase the lipid productivity up to 1.2 g L^−1^ h^−1^ [41]. Qiao et al*.* [172] stressed that likely the entire lipid, but also the glycolysis pathway need to be subjected to genetic manipulation to achieve the lipid productivities required for producing a commodity oil substitute [174, 297]. In recent TEAs the cost of microbial oils has been estimated at a lipid productivity of 0.13 g L^−1^ h^−1^ to 2.50 g L^−1^ h^−1^ (Table 2). It is generally recognised that for commercial production of a commodity oil substitute this value should be in the realistic range of 1 g L^−1^ h^−1^ to 2 g L^−1^ h^−1^ [49, 50, 229, 257, 296]. However, Caspeta and Nielsen [257] argued that only for lipid productivities as high as 7.5 g L^−1^ h^−1^, yeast lipids may be cost-competitive as biodiesel feedstock.

### Output characteristics

The output characteristics of the fermentation can facilitate downstream processing and therefore lower the overall production cost. For instance, high-density cultivation (Additional file 1: Table S4) leads to lower volumes in cell separation, but only a small fraction of oleaginous yeasts (7%) are cultured at cell densities above 50 g L^−1^ (Additional file 1: Fig. S10c). Meesters et al*.* emphasised that high lipid concentrations are required for low-cost lipid production [292], and researchers at NREL (2013) later suggested that a lipid concentration of over 90 g L^−1^ should be reached in a commercial process towards fuel production [296] (Fig. 7). Moreover, the secretion of lipids into the broth would facilitate subsequent lipid recovery such as through centrifugation [47, 48, 257]. In this respect, the oil recovery involving solvent extraction has been estimated to around 13% of the total yeast lipid production cost [265, 286].

### Biorefinery economics

In a biorefinery setting, it would be valuable to use a microbe, which can grow on a range of different feedstocks, especially when a second-generation feedstock is employed with a limited and variable availability. For instance, it was estimated that around 114,000 tonnes of yeast oil could be produced from starchy household wastes in the UK per year [49]. Oleaginous yeasts with a wide substrate spectrum include *C. oleaginosus* [28, 89, 115, 138] and *M. pulcherrima* [104, 112, 116], for instance. Combining the advantages of multiple yeasts could be a suitable option to fully valorise a substrate [298, 299]. The simultaneous use of multiple substrates, such as sugar- and fat-based, has also been shown to improve lipid production with some oleaginous yeasts including *C. oleaginosus* [163, 192, 193]. However, one of the key issues when using a range of different feedstocks and producing a (cheap) bulk product are the logistics [49].

Producing multiple metabolites can be beneficial [50, 226]. For example, the concept of a lignocellulosic SCO biorefinery has been proposed [123, 300]. Valuable co-products of an oleaginous yeast process can include 2-PE [104], carotenoids [179, 202, 301], cellulases [136], citrate [41, 165, 169], emulsifier [302], exopolysaccharides [301], gluconic acid [303], lipases [135, 136], polyols such as arabitol [54, 169], erythritol [169], mannitol [169], glycerol [54, 205] and xylitol [304], polyol esters [226], as well as spent biomass [302], some of which are produced by frequently used *Rhodotorula* species and *M. pulcherrima* (Table 1). The co-production of bioethanol and biodiesel through employing two yeasts is also conceivable [299]. Koutinas et al*.* estimated that if side streams such as spent biomass and other co-products were sold, the production cost of yeast lipids could be as low as US$ 1000 per tonne [49]. Parsons et al*.* furthermore suggested that lipid production may become economically feasible if the lipids became the secondary product to a low-molecular-weight fermentation molecule such as succinic acid [305], for instance at around 20% (w/w) lipid. Karamerou et al*.* demonstrated that in their hypothetical plant demonstrating the lowest possible lipid price, that being able to sell the spent yeast biomass at US$ 1.75 per kilogram reduced the cost of the lipid to approximately 0. Similarly if a small molecule, such as succinic acid was produced alongside the lipid, diverting 25% of the carbon away from lipid production, then the lipid price could be similarly reduced [285].

From these investigations it is clear that the co-production of a proteinous rich fraction alongside the lipid gives the biggest benefits to reducing the cost of the lipid [305]. Yeast protein composition is species-dependent but generally contains a wide range of essential amino acids, including lysine and methionine [117]. For the development of a product two approaches have been suggested. In the first, the spent biomass is used in vitro after extraction of the lipid fraction. An analysis of *Pichia guilliermondii* showed that the spent biomass contained 25% protein and 44% carbohydrate, suggesting some kind of animal feed application [302]. However, an assessment of *Y. lipolytica* (protein content of 47%) cultured on glycerol showed that while the proteinous biomass was shown to be highly digestible in animal trials, the protein contained a relatively low concentration of sulphur-containing amino acids limiting the nutritional value [282]. Due to their high nucleic acid content leading to uric acid overproduction, and lacking other nutrients such as vitamin B12, yeasts can generally only partially substitute traditional sources of animal feed. It is therefore likely that for an animal feed application the yeast species will have to be carefully selected for amino acid profile and alternative metabolites rather than just elevated lipid production. For higher value applications however, such as for human consumption, it is beneficial to have a protein enhanced fraction, or simply a purified amino acid mixture.

### Consumer acceptance and regulation

A microbial oil would likely be accepted by consumers as a sustainable alternative to fossil or vegetable oils, if produced by non-GM and non-pathogenic yeast, which have been used in food biotechnology for centuries. Moreover, microbial specialty oils are already on the market [33]. Though in a recent episode of the Netflix programme ‘Grace and Frankie’ (Series 2 Episode 10), the main protagonists, in searching for a palm oil substitute negatively link a yeast lipid product to a *Candida albicans* infection. This demonstrates that even with a non-GMO route, in certain fields there is likely to be some consumer resistance. Therefore, some degree of uncertainty would still be associated with a launch of a commodity oil substitute [33].

Microbial oils derived from GMOs, such as speciality oils, however, suffer from regulatory hurdles and weak consumer perception in some regions, particularly for food purposes. Indeed, in many developed countries the public perception on eating GM food is mainly negative. In China, for example, around 47% of consumers have a negative view on GM food [263]. The Eurobarometer (2010) revealed “overall suspicion of GM food amongst the European public” [262]. Around 59% of Europeans do not consider GM food safe for their own and their family's health.

In terms of approval, this of GMO-derived food is more complex and depends on national regulation. With the EU pursuing a “precautionary approach” for bringing food-grade lipids to the market, Regulation (EC) No 1829/2003 on GM food and feed imposes hurdles requiring pre-market authorisation. Additional uncertainty was added in 2015, since when individual member states can opt out on use of GM food. In the USA and China, on the other hand, GM foods have to climb less hurdles compared to Europe [306].

## Sustainability of yeast lipid production

Due to the lack of industrial data on the process, only a handful of life cycle assessments have been published around the production of oleaginous yeasts. Some of the key requirements are the sustainability of the feedstock, with a key focus on waste resources over first generation sugars. Another key factor is the nature of an aerobic fermentation, as vast amounts of CO_2_, typically accounting for around 35% (w/w) to 50% (w/w) of the feedstock carbon [13, 54], are released into the environment. With a moderate lipid yield of 0.20 g g^−1^ from glucose (Additional file 1: Fig. S10), this means that per kilogram of oil produced, up to 3.65-kg CO_2_ are be produced through the yeast alone. The total greenhouse gas emission of SCO production from glucose are in the range of 7.2 to 11.6 kg CO_2_-equiv. kg^−1^ oil with energy requirements of the fermentation and feedstock procurement largely contributing [52]. This compares to 2.8 to 19.7 kg CO_2_-equiv. kg^−1^ to produce palm oil in South Asia [307]. The CO_2_ balances can be remedied through the feedstock, for example when utilising lignocellulosic biomass [116] or macroalgae [104]. Although the production of yeast lipids from macroalgae would still be associated with around 2.5 to 9.9 kg CO_2_-equiv. kg^−1^ refined oil [51], this could potentially be lower compared to terrestrial oil production [307]. An intelligent solution including carbon capture could be the integration of a yeast and autotrophic algae lipid production process, in which the algae utilise the CO_2_ produced by the yeast [308, 309]. The acidification (0.004 kg SO_2_-equiv. kg^−1^ oil using sugarcane as feedstock) and eutrophication potential (0.007 kg PO_4_-equiv. kg^−1^ oil) of yeast lipid production may be reduced compared to crop oil production depending on the fermentation feedstock [52].

For feedstock procurement, fermentation and refining of lipids, vast amounts of water are also required [51]. Processing options to minimise water usage include high-density flow operation [28, 54], broth recycle [310, 311], cultivation in seawater [164, 312], or using a thermotolerant strain [232]. Furthermore, for extracting the lipids from the yeast, large amounts of toxic organic solvents, estimated around 40 kg hexane per tonne of oil extracted [49], are typically required. An alternative could be the use of green solvents, but their cost is still too high for commercial viability [313]. Moreover, the extracellular secretion of lipids [47, 48] or using whole cell biomass as the product [12, 258] may allow a solvent step to be removed entirely [257]. The full utilisation of the oleaginous yeast product suite, especially the protein fraction, can also benefit the environmental impact, for example through energy generation on-site [305]. Typically many life cycle assessment studies have employed a biorefinery approach to SCO production. Of the many co-product options, the protein fraction appears to play the greatest role in determining viability, with co-product strategy crucial to reducing the environmental impact of SCO production to be equivalent or lower with typical terrestrial plant oils [305].

If environmental and social benefits were clearly demonstrated, the potential sustainability of a yeast-derived commodity oil substitute could benefit the process economics through a higher value for costumers [268, 291]. In this respect, certification of a more sustainable product is also conceivable for biofuels [314]. Successful marketing could therefore be one of the key elements to producing yeast lipids on an industrial scale. However, with a small price premium unlikely to be sufficient for the yeast lipids to be produced economically, subsidies, such as the case of first- and second-generation biodiesel [38], would be required for the current technology to be cost-competitive [257]. To produce SCOs sustainably as a commodity oil, governmental but ultimately international market interventions towards social or environmental benefits, with “socio-economic justice as a core value” [315], rather than solely energy security, are inevitable [291, 314]. Finally, there is a possibility of carbon taxation being levied on agricultural products with a high carbon cost such as palm oil from deforested land [291, 307], which could aid further SCO development.

## Conclusions

Substantial progress has been made in developing processes featuring oleaginous yeasts, particularly in the last decade, which saw a ten-fold increase in annual yeast lipid publications. Remarkably, lipid productivities over 1.0 g L^−1^ h^−1^ have been achieved with non-GM yeasts, and lipid production uncoupled from nutrient limitation and cell growth through lipid secretion. Emerging techno-economic analyses and life-cycle assessments have further advanced the credibility of the yeast lipid concept. However, the lipid production costs are the biggest hurdle towards their commercialisation as a sustainable alternative to commodity oils such as palm oil or derivatives such as biodiesel; and low consumer acceptance of a GMO-derived oil hampers the commercialisation of speciality oils. Therefore, the commercialisation of a yeast lipid is contingent upon further progress in process and product design, marketing and market intervention:

1. Process design: to produce a commodity oil substitute using a sustainable yet cheap feedstock and robust oleaginous yeast, and achieving high production rates are inevitable. Therefore, researchers should continue pushing the boundaries of already discovered native and promising yeasts such as *Cutaneotrichosporon oleaginosus*. To this end, the continuous extracellular lipid production may be a promising scenario for low-cost lipid production achieving high lipid yields. The isolation of new oleaginous species only appears to be beneficial towards industrial lipid production if they are unusually composed, produce valuable secondary metabolites or are capable of secreting their lipids.
2. Product design: the product suite of oleaginous yeasts must be effectively used. This specifically includes the yeast biomass for human or animal nutrition, for which solvent contamination could be avoided through extracellular lipid production or using the whole cell biomass as a product. Moreover, the valuation of secondary metabolites can facilitate economic production of yeast lipids, but policies might be required to avoid shifting the metabolic flux towards a more valuable product. Conceivable is also a process, in which the lipid is the co-product produced in lower quantities along with a low-molecular-weight fermentation molecule such as succinic acid.
3. Marketing: the product must be effectively marketed. The yeast biomass and oil could have superior characteristics such as being tuneable and more sustainable, leading to an increased value, potentially sufficient for economic production. Furthermore, the public perception of GMOs remains a major challenge. Modified yeasts typically achieve advanced production parameters and could also deliver speciality oils, an economically attractive proposition, through the targeted alteration of the fatty acid profile.
4. Intervention: for economically producing a commodity oil substitute with the current technology at current oil prices, likely governmental but ultimately international market interventions are required. Directives such as the revised Renewable Energy Directive (RED II) by the EU could increase the potential for yeast lipid to be incorporated into the transportation fuel portfolio, for example, but for a sustainable oil such interventions should have global social and environmental benefits as core values. Finally, potential carbon taxation on agricultural products such as palm oil may aid further development of SCO processes.

The history of oleaginous yeast research has shown that oleaginous yeast processes have been intensively investigated and brought to scale in times of crisis and uncertainty. With climate change, depletion of fossil resources and ecological damage effecting global food supply chains, yeast lipids could play a vital role in multiple areas in the twenty-first century. Indeed, in light of the predicted shortage of fossil fuels, a possible ban on using fossil resources to mitigate climate change, the growing population and consequently further strain on producing edible oils in direct competition with pristine forests, it is vital that microbial processes are developed, scaled and ready to produce lipids on the industrial scale in the short to medium term.

## Supplementary Information

**Additional file 1.** A supplementary information containing methods used in the data collection, further information on publishing trends within this data, and published processing conditions, lipid productivity and total lipid contents of the oleaginous yeasts discussed in this review.

## Data Availability

All data and material used for preparing the manuscript appear in the submitted article.

## Footnotes

1 Presumably, this number is lower compared to the fraction of yeast being capable of producing over 20% (w/w) lipid due to constraints in the screening technique such as using a single feedstock and fixed operation conditions, but also because for several species, such as *S. cerevisiae*, only certain strains have been reported oleaginous (Additional file 1: Table S3).
