# Supplementary material for: The history, state of the art and future prospects for oleaginous yeast research
Source: Microb Cell Fact. 2021 Dec 7;20:221. doi: 10.1186/s12934-021-01712-1 (PMC8650507; doi:10.1186/s12934-021-01712-1)
Supplement: Supplementary file 1 — Additional file 1. A supplementary information containing methods used in the data collection, further information on publishing trends within this data, and published processing conditions, lipid productivity and total lipid contents of the oleaginous yeasts discussed in this review. [file 12934_2021_1712_MOESM1_ESM.docx]

# Title page

**Additional information for**

**The history, state of the art and future prospects for oleaginous yeast research**

Felix Abeln^a, b, *^, Christopher J. Chuck^a, *^

^a^ Department of Chemical Engineering, University of Bath, BA2 7AY, Bath, UK

^b^ Centre for Sustainable and Circular Technologies, University of Bath, BA2 7AY, Bath UK

^*^ Correspondence to: felix.abeln@bath.edu; c.chuck@bath.ac.uk

**This file includes on 22 pages (please see navigation pane):**

Methods

Table S1 to S5

Figures S1 to S10

References

# Methods

Methods

Abstract and citation database Scopus was used for the search of scientific research publications. For their total number, the search was limited to document type article, article in press, conference paper and letter, language English and year from 1975. This year was chosen, as 1972 the term ‘oleaginous yeast’ was created (1) and the English language used in the majority of oleaginous yeast publications. For those concerning oleaginous yeasts, the additional search terms were (yeast AND oleaginous) OR (yeast AND lipid AND content) OR (oleaginous AND lipid) OR (single cell oil AND yeast) OR (microbial oil AND yeast) to be in the title, abstract or keywords. The search output was screened for those involving yeast fermentation, with a lipid content reported in text form of at least 20% (w/w). Accordingly, an average of 11 ± 8% of the search output were selected. Additionally, oleaginous yeast publications were searched for through reference lists of the selected publications and relevant reviews to account for those not meeting the search criteria or not available on Scopus. A total of 684 research publications from 1975 up to 2020 were selected as concerning oleaginous yeasts and used for data analysis. Including the oleaginous yeast publications prior to 1975, of which several are covered in the section ‘Industrial development and key research for oleaginous yeasts’ of the manuscript and by Woodbine (2), and those missed by the above criteria such as those in languages other than English, the total number of oleaginous yeast research publications can be estimated as approaching 1,000 currently.

For the data analysis, multiple strains of the same species were counted in a single publication when they were wild type strains, but for genetically modified versions (genetically engineered, laboratory evolved or mutated) from the same wild type, only this with the highest lipid content was counted. Per counted organism and publication, the highest lipid content, yield, productivity, concentration, cell density and working volume were recorded. Only data from organisms with an identified species name was recorded. For fed-batch cultures, the working volume represents the initial working volume. If only the reactor volume was given, the working volume was assumed as 20% (w/w) reactor volume for flask cultures and 70% (w/w) for each stirred tank reactor, airlift and bubble column. Publications, in which the results are clearly not repeatable, such as yields higher than the theoretical on pure saccharides, were neglected. Per selected publication, the proposed applications were categorised into the four categories biofuel, food/supplement, animal feed and (other) oleochemicals. Per selected publication, all carbon sources of the main culture medium for producing lipid were recorded and categorised (see Figure 3 of the manuscript). If the type of glycerol was not specified, this was assumed as pure. Autotrophic co-cultures with algae where the origin of the lipids is not clear were not included. Statistical errors were calculated as standard deviation.

# Table S1

**Table S1:** Typical fatty acid composition of selected vegetable oils and fats. Depicted fatty acids are palmitic (16:0), oleic (18:1), stearic (18:0), linoleic (18:2), alpha/gamma-linolenic (18:3), paullinic (20:1) and heneicosenoic (C21:1) acid. Values are given in % (w/w) and a hyphen indicates values < 1% (w/w). Other fatty acids may be present, but only in minor amounts. Data obtained from (3).

| **Oil or fat** | **Fatty acid composition** | | | | | | | **Saturated fatty acids** |
| --- | --- | --- | --- | --- | --- | --- | --- | --- |
|  | C16:0 | C18:0 | C18:1 | C18:2 | C18:3 | C20:0 | C21:1 |  |
| Rapeseed oil ^a^ | 4 | 2 | 56 | 26 | 10 | - | 2 | 6 |
| Sunflower oil ^b^ | 6 | 5 | 20 | 69 | - | - | - | 11 |
| Olive oil | 10 | 2 | 78 | 7 | 1 | - | - | 12 |
| Soybean oil | 11 | 4 | 22 | 53 | 8 | - | - | 15 |
| Groundnut oil | 13 | 3 | 38 | 41 | - | - | - | 16 |
| Cottonseed oil | 27 | 2 | 18 | 51 | - | - | - | 29 |
| Palm oil | 44 | 4 | 40 | 10 | - | - | - | 48 |
| Cocoa butter | 25 | 35 | 35 | 3 | - | 1 | - | 60 |

^a^ low-erucic (canola oil)

^b^ high-linoleic (regular)

# Table S2

**Table S2:** The further confirmed oleaginous yeasts. Displayed are yeast or yeast-like species which have been identified as oleaginous in two publications (according to the same criteria as stated in the caption of Figure 5 of the manuscript), and their average lipid content. The full methodology used to collect and analyse the presented data is given at the beginning of this document.

| **Current species name** | Lipid content (% w/w) |
| --- | --- |
| *Blastobotrys adeninivorans*  *Candida freyschussii*  *Candida viswanathii*  *Cryptococcus ramirezgomezianus*  *Cyberlindnera fabianii*  *Cystobasidium laryngis*  *Cystobasidium oligophagum*  *Kazachstania spencerorum*  *Lipomyces doorenjongii*  *Lipomyces kockii*  *Lipomyces mesembrius*  *Moesziomyces parantarcticus*  *Naganishia adeliensis*  *Naganishia vishniacii*  *Pichia manshurica*  *Rhodosporidiobolus colostri*  *Rhodosporidiobolus lusitaniae*  *Rhodotorula taiwanensis*  *Solicoccozyma aeria*  *Sporobolomyces pararoseus*  *Trichosporon coremiiforme*  *Vanrija musci*  *Vishniacozyma heimaeyensis*  *Vishniacozyma psychrotolerans*  *Waltomyces lipofer*  *Wickerhamomyces anomalus*  *Yarrowia galli*  *Yarrowia oslonensis* | 20.0  33.5  55.2  31.8  29.3  31.1  46.0  37.4  68.8  49.2  32.7  46.6  37.0  52.9  46.5  44.9  41.1  34.5  56.6  55.3  38.2  39.0  45.2  46.2  32.1  23.5  34.4  24.9 |

# Table S3

**Table S3:** The further identified oleaginous yeasts. Displayed are yeast or yeast-like species which have been identified as oleaginous in a single publication (according to the same criteria as stated in the caption of Figure 5 of the manuscript), and their lipid content.

| **Current species name** | Lipid content (% w/w) | **Current species name** | Lipid content (% w/w) | **Current species name** | Lipid content (% w/w) |
| --- | --- | --- | --- | --- | --- |
| *Apiotrichum dulcitum* | 28.7 | *Hannaella aff. zeae* | 23.1 | *Saturnispora silvae* | 30.1 |
| *Apiotrichum laibachii* | 21.9 | *Kurtzmaniella quercitrusa* | 30.3 | *Scheffersomyces segobiensis* | 24.6 |
| *Apiotrichum loubieri* | 34.9 | *Leucosporidium creatinivorum* | 55.1 | *Scheffersomyces stipitis* | 26.0 |
| *Aureobasidium melanogenum* | 66.3 | *Leucosporidium scottii* | 48.6 | *Schwanniomyces occidentalis var. occidentalis* | 41.9 |
| *Blastobotrys raffinosifermentans* | 38.1 | *Lindnera saturnus* | 30.0 | *Solicoccozyma phenolica* | 51.7 |
| *Candida haemulonis* | 33.0 | *Lipomyces orientalis* | 56.4 | *Sporobolomyces aff. beijingensis* | 48.3 |
| *Candida hispaniensis* | 67.1 | *Lipomyces yamadae* | 29.4 | *Sporobolomyces aff. inositophilus* | 45.9 |
| *Candida holmii* | 32.3 | *Lipomyces yarrowii* | 49.4 | *Sporobolomyces bannaensis* | 34.1 |
| *Candida lambica* | 34.8 | *Macalpinomyces spermophorus* | 30.3 | *Sporobolomyces carnicolor* | 54.5 |
| *Candida membranaefaciens* | 29.6 | *Myxozyma melibiosi* | 23.4 | *Sporobolomyces johnsonii* | 34.1 |
| *Candida parapsilosis* | 24.2 | *Myxozyma mucilagina* | 20.8 | *Sporobolomyces metaroseus* | 34.6 |
| *Candida pseudolambica* | 35.3 | *Naematelia encephala* | 41.7 | *Sporobolomyces salmonicolor* | 36.0 |
| *Candida rugosa* | 38.5 | *Naganishia diffluens* | 23.2 | *Sporobolomyces shibatanus* | 20.0 |
| *Candida tenuis* | 25.6 | *Naganishia globosa* | 27.7 | *Starmera amethionina* | 58.6 |
| *Candida utilis* | 24.0 | *Naganishia uzbekistanensis* | 34.4 | *Starmerella bombicola* | 21.1 |
| *Cryptococcus magnus var. magnus* | 21.2 | *Nakazawaea molendinolei* | 50.0 | *Sterigmatosporidium polymorphum* | 20.9 |
| *Cyberlindnera saturnus* | 25.2 | *Occultifur externus* | 57.0 | *Torulaspora maleeae* | 50.7 |
| *Cystofilobasidium capitatum* | 22.6 | *Ogataea angusta* | 34.9 | *Trichosporon domesticum* | 33.0 |
| *Cystofilobasidium macerans* | 42.1 | *Papiliotrema flavescens* | 22.6 | *Trichosporon fermentans* | 20.0 |
| *Farysia acheniorum* | 31.2 | *Phaffia rhodozyma* | 40.2 | *Trichosporon ovoides* | 31.6 |
| *Filobasidium cf. uniguttulatum* | 20.3 | *Pichia occidentalis* | 28.9 | *Trichosporon porosum* | 34.1 |
| *Filobasidium globisporum* | 20.3 | *Pseudohyphozyma bogoriensis* | 29.2 | *Trigonosporomyces aff. hylophilus* | 20.2 |
| *Filobasidium inconspicuum* | 29.3 | *Pseudozyma hubeiensis* | 24.6 | *Vishniacozyma victoriae* | 22.2 |
| *Filobasidium magnum* | 25.7 | *Rhodosporidiobolus odoratus* | 47.7 | *Wickerhamomyces siamensis* | 27.0 |
| *Filobasidium oeirense* | 25.8 | *Rhodosporidiobolus poonsookiae* | 50.4 | *Yarrowia alimentaria* | 42.8 |
| *Filobasidium wieringae* | 52.7 | *Rhodotorula mucilaginosa* | 23.4 | *Yarrowia deformans* | 34.4 |
| *Goffeauzyma gastrica* | 20.5 | *Rhodotorula araucariae* | 60.1 | *Yarrowia hollandica* | 50.7 |
| *Hamamotoa singularis* | 23.8 | *Robbauera albescens* | 22.9 | *Yarrowia keelungensis* | 22.5 |
|  |  |  |  | *Yarrowia yakushimensis* | 45.3 |

# Table S4

**Table S4:** High-density oleaginous yeast cultures. Listed are oleaginous yeasts cultured at cell densities above 100 g L^−1^ and the corresponding process characteristics.

| **Yeast species** | **Strain *** | **Carbon source** | **Operation modes** | **V_w_**  **(L)** | **X_max_**  **(g L^−1^)** | **L_max_**  **(g L^−1^)** | **Y_L_**  **(g g^−1^)** | **P_L_**  **(g L^−1^ h^−1^)** | **Year** | **Ref.** |
| --- | --- | --- | --- | --- | --- | --- | --- | --- | --- | --- |
| *Cutaneotrichosporon oleaginosus* | ATCC 20509 | Glycerol | Batch, fed-batch | 1 | 118.0 | 29.5 | 0.11 | 0.59 | 1996 | (4) |
| *Cutaneotrichosporon oleaginosus* | ATCC 20509 | Acetate, volatile fatty acids from anaerobic digestion | Batch, fed-batch, continuous | 5 | 168.0 | 126.0 | N/M | 0.66 | 2011 | (5) |
| *Cutaneotrichosporon oleaginosus* | O3 (EV) | Glucose | Batch, fed-batch, continuous | 14 | 104.1 | 86.1 | 0.25 | 0.50 | 2011 | (6) |
| *Cutaneotrichosporon oleaginosus* | ATCC 20509 | Glucose | Batch, fed-batch | 6 | 132.0 | 71.3 | 0.24 | 0.08 | 2017 | (7) |
| *Lipomyces starkeyi* | AS 2.1560 | Glucose | Batch, two-stage batch | 10 | 104.6 | 67.9 | N/M | 1.60 | 2011 | (8) |
| *Lipomyces starkeyi* | DSM 70296 | Glucose, starch hydrolysate | Batch, fed-batch | 1.5 | 109.8 | 64.5 | N/M | 0.40 | 2014 | (9) |
| *Metschnikowia pulcherrima* | N/M | Distillery wastewater | N/M | 3.5 | 115.0 | 41.4 | 0.16 | 0.35 | 2019 | (10) |
| *Metschnikowia pulcherrima* | NCYC 4331 (EV) | Glucose | Batch, fed-batch, semi-continuous, repeated batch, continuous | 2 | 122.6 | 50.6 | 0.16 | 0.18 | 2019 | (11) |
| *Rhodotorula glutinis* | Rh-00301 | Sucrose | Batch, fed-batch | 2 | 106.0 | 66.8 | 0.18 | 0.84 | 2017 | (12) |
| *Rhodotorula toruloides* | NRRL Y-1091 | Glucose | Fed-batch | 2 | 185.0 | 74.0 | N/M | 0.88 | 1986 | (13) |
| *Rhodotorula toruloides* | Y4 (EV) | Glucose | Batch, fed-batch | 7 | 151.5 | 72.7 | 0.23 | 0.54 | 2007 | (14) |
| *Rhodotorula toruloides* | Y4 (EV) | Tuber aqueous extract | Batch, fed-batch | 9 | 113.0 | 45.4 | N/M | 0.38 | 2010 | (15) |
| *Rhodotorula toruloides* | Y4 (EV) | Glucose | Fed-batch, repeated fed-batch | 9 | 127.5 | 78.8 | 0.24 | 0.57 | 2011 | (16) |
| *Rhodotorula toruloides* | Y4 (EV) | Glucose | Fed-batch | 9 | 120.8 | 76.1 | N/M | N/M | 2012 | (17) |
| *Rhodotorula toruloides* | CECT 1137 | Glucose | Fed-batch | 14 | 144.0 | 103.7 | 0.23 | 1.48 | 2013 | (18) |
| *Rhodotorula toruloides* | CECT 1137 | Glucose | Batch, fed-batch | 8 | 110.0 | 70.4 | N/M | 1.05 | 2014 | (19) |
| *Rhodotorula toruloides* | Y4 (EV) | Glucose | Batch, fed-batch | 7 | 100.0 | 70.0 | 0.20 | 0.52 | 2015 | (20) |
| *Rhodotorula toruloides* | RT880-ADS (GE) | Glucose | Batch, fed-batch | 0.4 | 118.4 | 89.4 | 0.22 | 0.62 | 2016 | (21) |
| *Yarrowia lipolytica* | NS432 (GE) | Glucose | Batch, fed-batch | 1 | 115.8 | 84.5 | 0.20 | 0.73 | 2016 | (22) |
| *Yarrowia lipolytica* | ADgm-hi (GE) | Glucose | Batch, fed-batch | 1.6 | 148.0 | 98.9 | 0.27 | 1.20 | 2017 | (23) |
| *Yarrowia lipolytica* | MTYL065 (GE) | Acetate | Semi-continuous | 1.5 | 194.0 | 115.0 | 0.16 | 0.80 | 2017 | (24) |

* EV, evolved; GE, genetically engineered.

Abbreviations: V_w_, working volume (initial working volume for fed-batch); X_max_, maximum dry cell weight; L_max_, maximum lipid concentration; Y_L_, lipid yield; P_L_, lipid productivity; N/M, not mentioned.

# Table S5

**Table 5:** Average lipid productivities of the most prominent and promising oleaginous yeasts. The data is based on oleaginous yeast research from 1975 to 2020 (684 publications). The full methodology used to collect and analyse the presented data is given at the beginning of this document.

| **Yeast species** | **Average lipid productivity (g L^−1^ h^−1^)** | |
| --- | --- | --- |
|  | Only native strains | All strains |
| *Yarrowia lipolytica* | 0.08 ± 0.10 | 0.13 ± 0.21 |
| *Rhodotorula toruloides* | 0.12 ± 0.22 | 0.14 ± 0.22 |
| *Cutaneotrichosporon oleaginosus* | 0.15 ± 0.17 | 0.16 ± 0.17 |
| *Lipomyces starkeyi* | 0.14 ± 0.25 | 0.14 ± 0.24 |
| *Rhodotorula glutinis* | 0.11 ± 0.16 | 0.10 ± 0.16 |
| *Rhodotorula mucilaginosa* | 0.09 ± 0.08 | 0.09 ± 0.08 |
| *Trichosporon cutaneum* | 0.07 ± 0.05 | 0.07 ± 0.05 |
| *Metschnikowia pulcherrima* | 0.08 ± 0.10 | 0.09 ± 0.10 |

# Figure S1


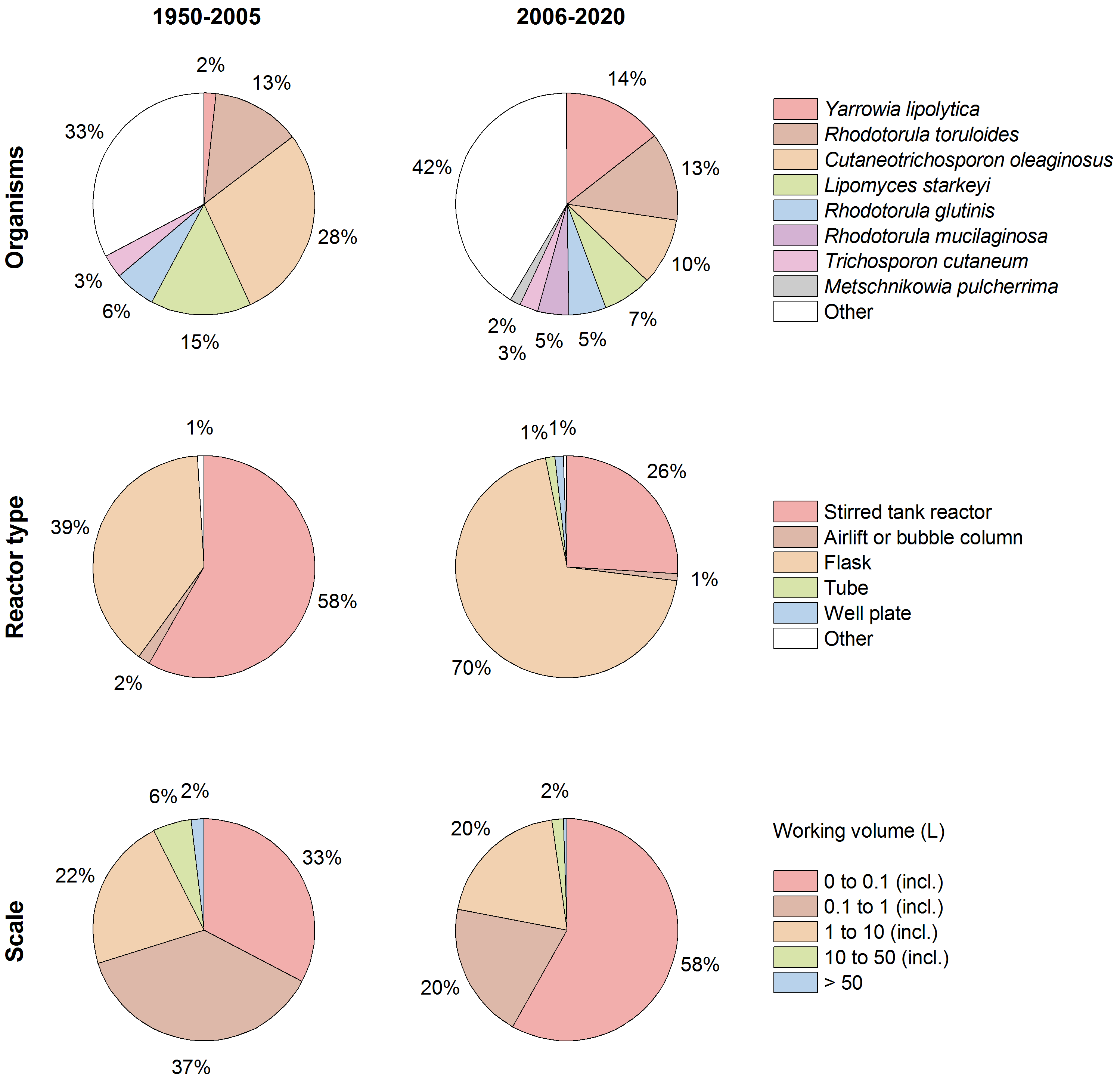


**Figure S1:** Distribution of oleaginous yeast (OY) species, reactor types and scales used at different times in history. Depicted is the percentage of an OY species with respect to all OYs cultured; the percentage of OYs cultured in a specific reactor type; and those cultured at a specific scale – during 1950 to 2005 or 2006 to 2020. *Cutaneotrichosporon oleaginosus* (discovered in 1978 (25)) was particularly popular during the 1980s, when the production of a cocoa butter equivalent was attempted (26). *Yarrowia lipolytica*, on the other hand, gained increased interested more recently due to advanced development of genetic tools. The labels for values < 1% are not displayed. The full methodology used to collect and analyse the presented data is given at the beginning of this document.

# Figure S2


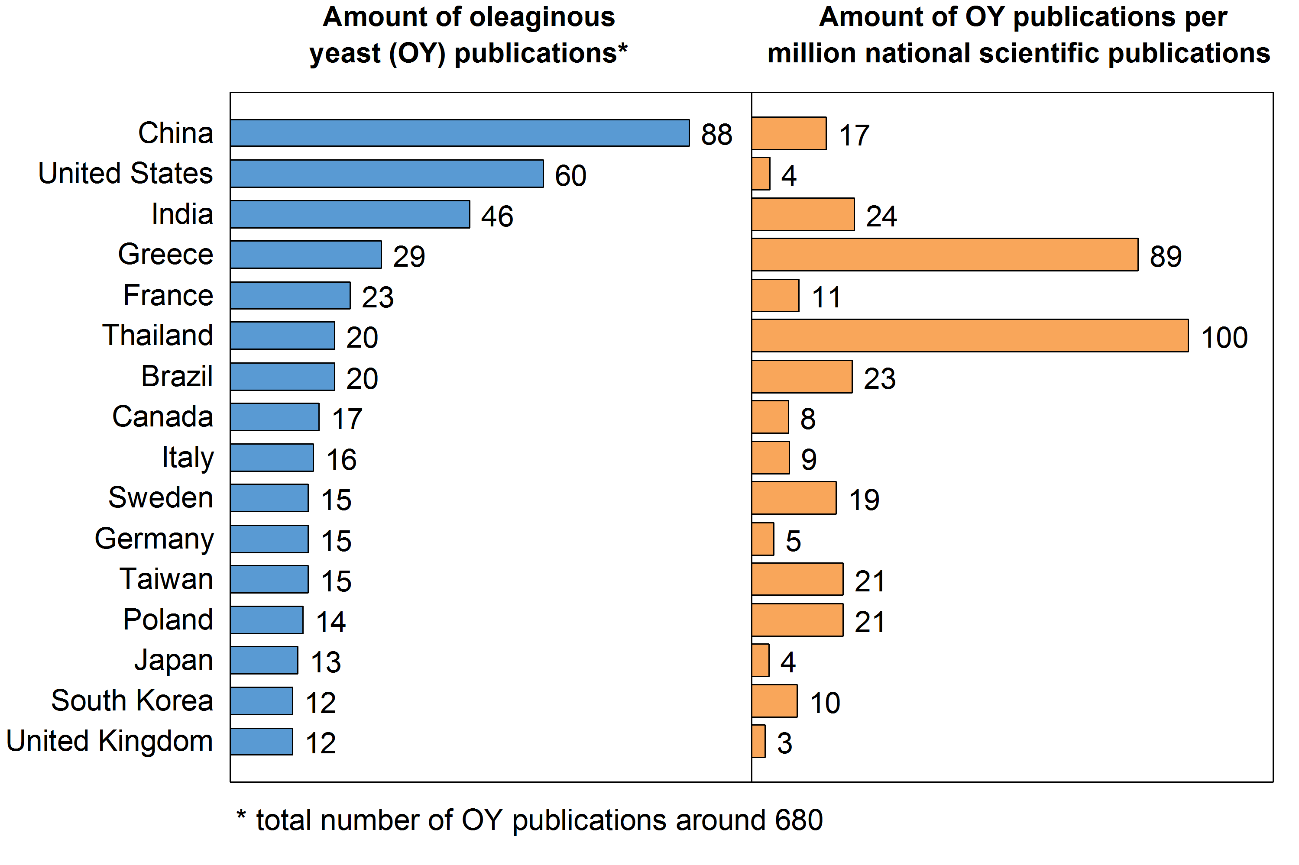


**Figure S2:** Countries most active in oleaginous yeast (OY) research. Depicted are the total amount of OY publications associated to a country, as well as those with respect to the countries’ overall scientific output. One publication may be associated to multiple countries if authors from multiple countries (according to the author affiliation) were involved. The full methodology used to collect and analyse the presented data is given at the beginning of this document.

# Figure S3


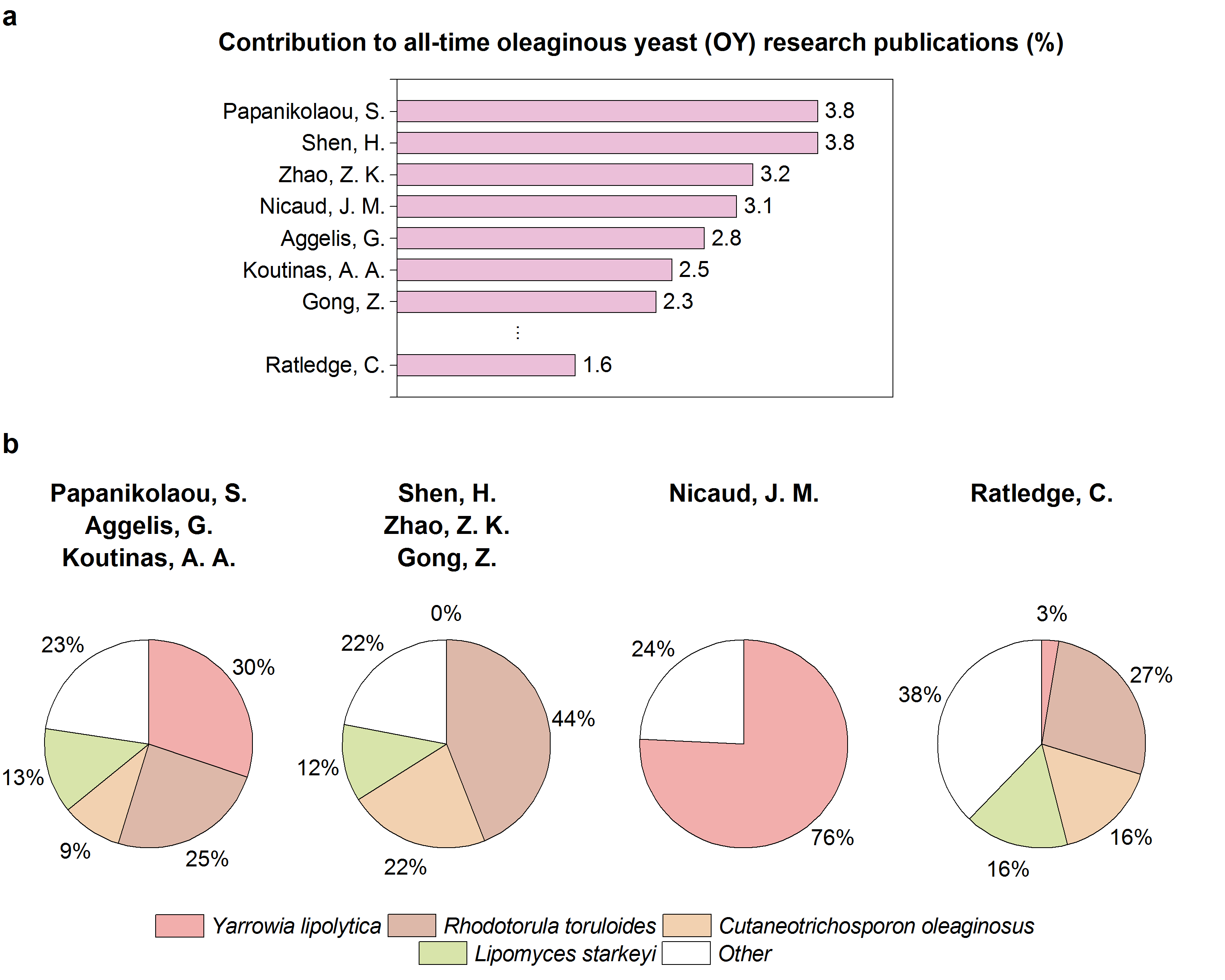


**Figure S3:** Most influential authors in oleaginous yeast (OY) research. (a) Listed are the authors of publications concerning OYs with a lipid content reported larger and equal to 20% (w/w). (b) Listed are often-collaborating YL researchers and their most frequently used OY species. The full methodology used to collect and analyse the presented data is given at the beginning of this document.

# Figure S4


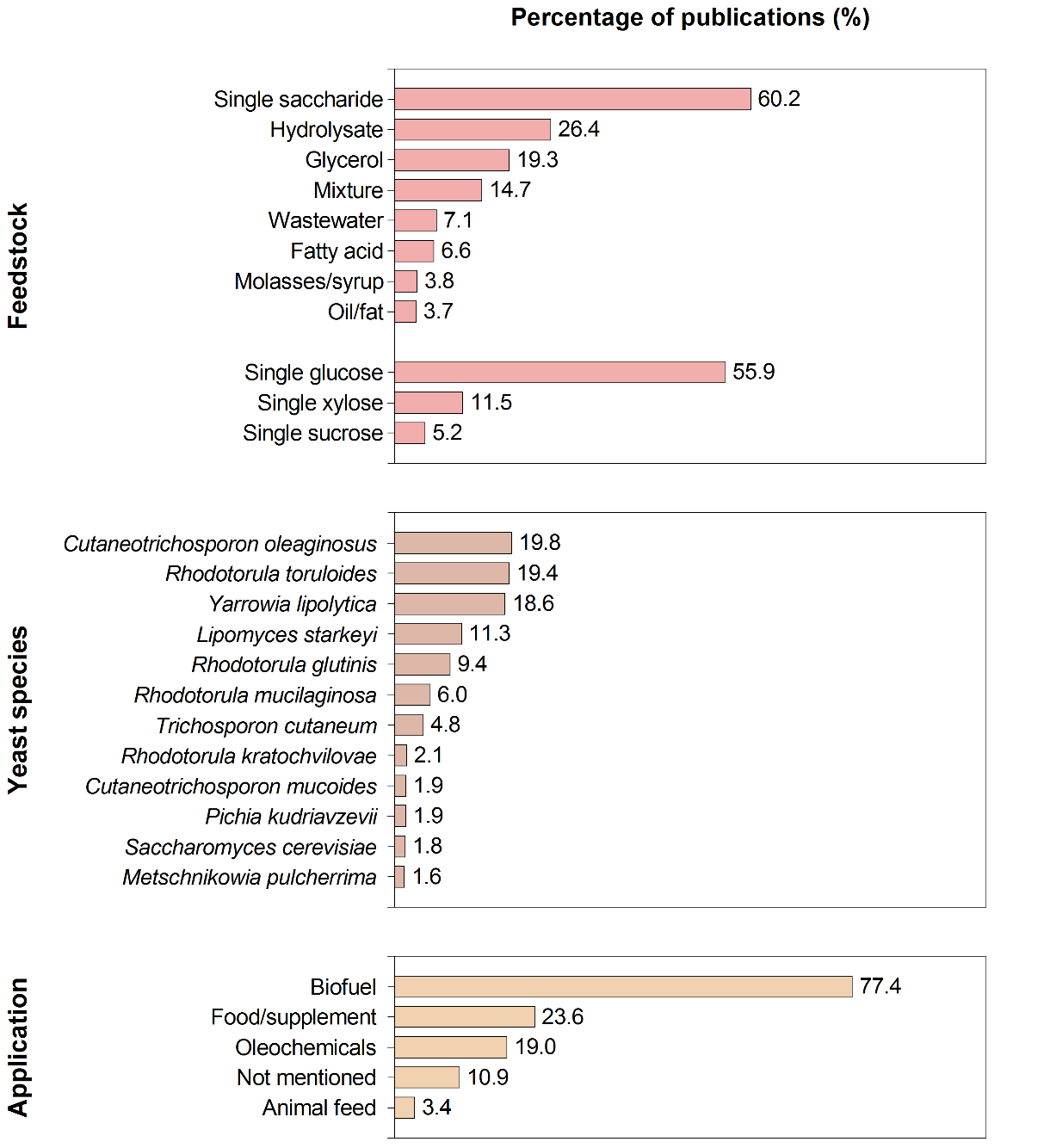


**Figure S4:** Percentages of oleaginous yeast publications using a certain feedstock, yeast species or proposing a specific application. Please see Figure 3 of the manuscript for details of the carbon sources comprising each category. The full methodology used to collect and analyse the presented data is given at the beginning of this document.

# Figure S5


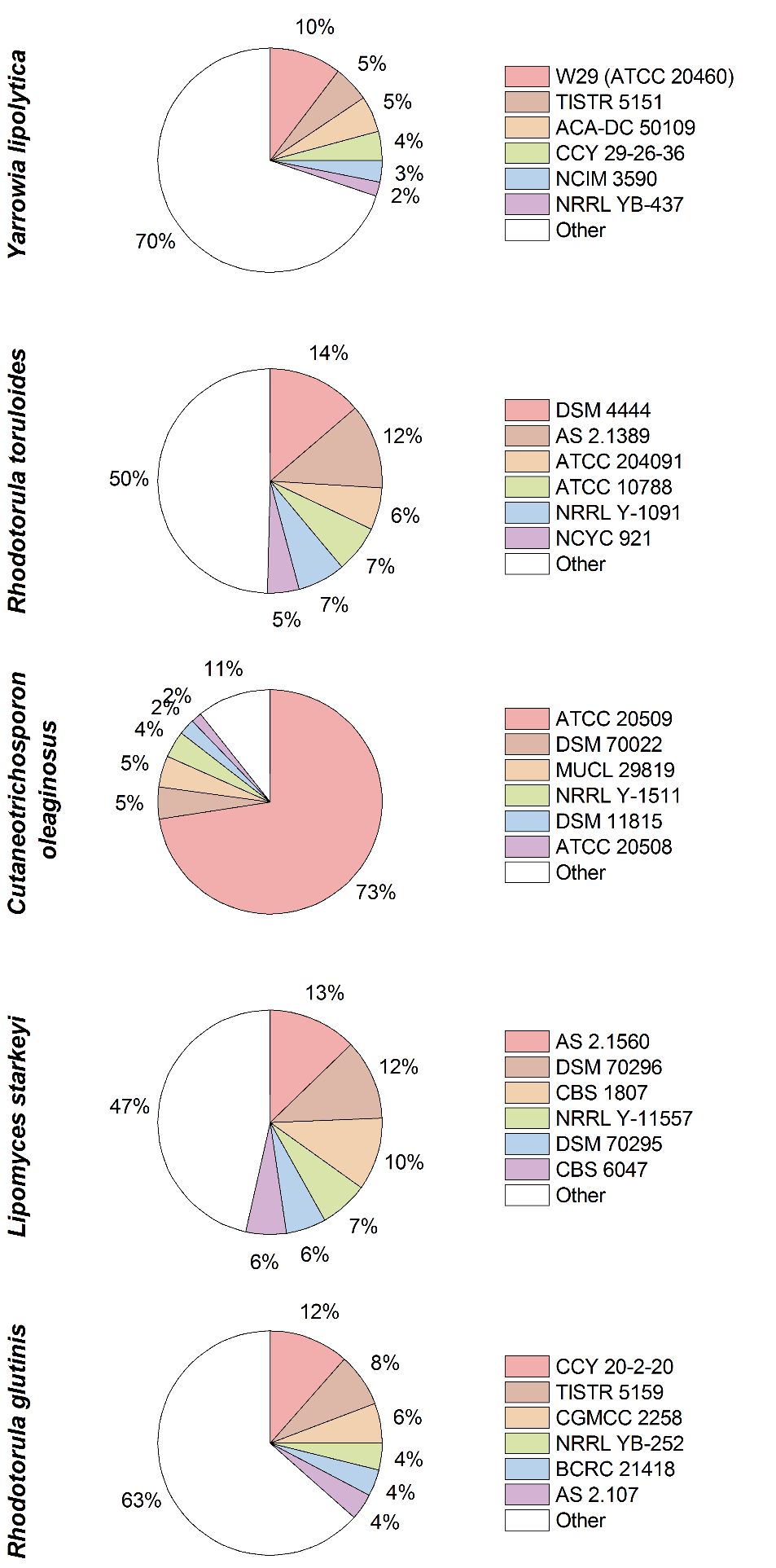


**Figure S5:** The most used native strains of the most prominent oleaginous yeasts (OYs). Displayed is the percentage of a strain used in relation to the species. The category ‘other’ includes lesser used strains as well as those not deposited. Per publication, multiple strains of the same species were counted (for wild-type strains only). The culture collections are ACA-DC, Agricultural College of Athens - Dairy Collection (Greece); AS, China General Microbiological Culture Collection Center (China); ATCC, American Type Culture Collection (USA); BCRC, Bioresource Collection and Research Center (Taiwan); CBS, Central Bureau of Fungal Cultures (Netherlands); CCY, Culture Collection of Yeasts (Slovakia); CGMCC, China General Microbiological Culture Collection Center (China); DSM, German Collection of Microorganisms and Cell Cultures (Germany); MUCL, Agro-food & Environmental Fungal Collection (Belgium); NCIM; National Collection of Industrial Microorganisms (India); NCYC, National Collection of Yeast Cultures (UK); NRRL, Agricultural Research Service (Northern Regional Research Laboratory, USA); TISTR, Thailand Institute of Scientific and Technological Research (Thailand). The same organism may have been deposited in different depositories. The full methodology used to collect and analyse the presented data is given at the beginning of this document.

# Figure S6


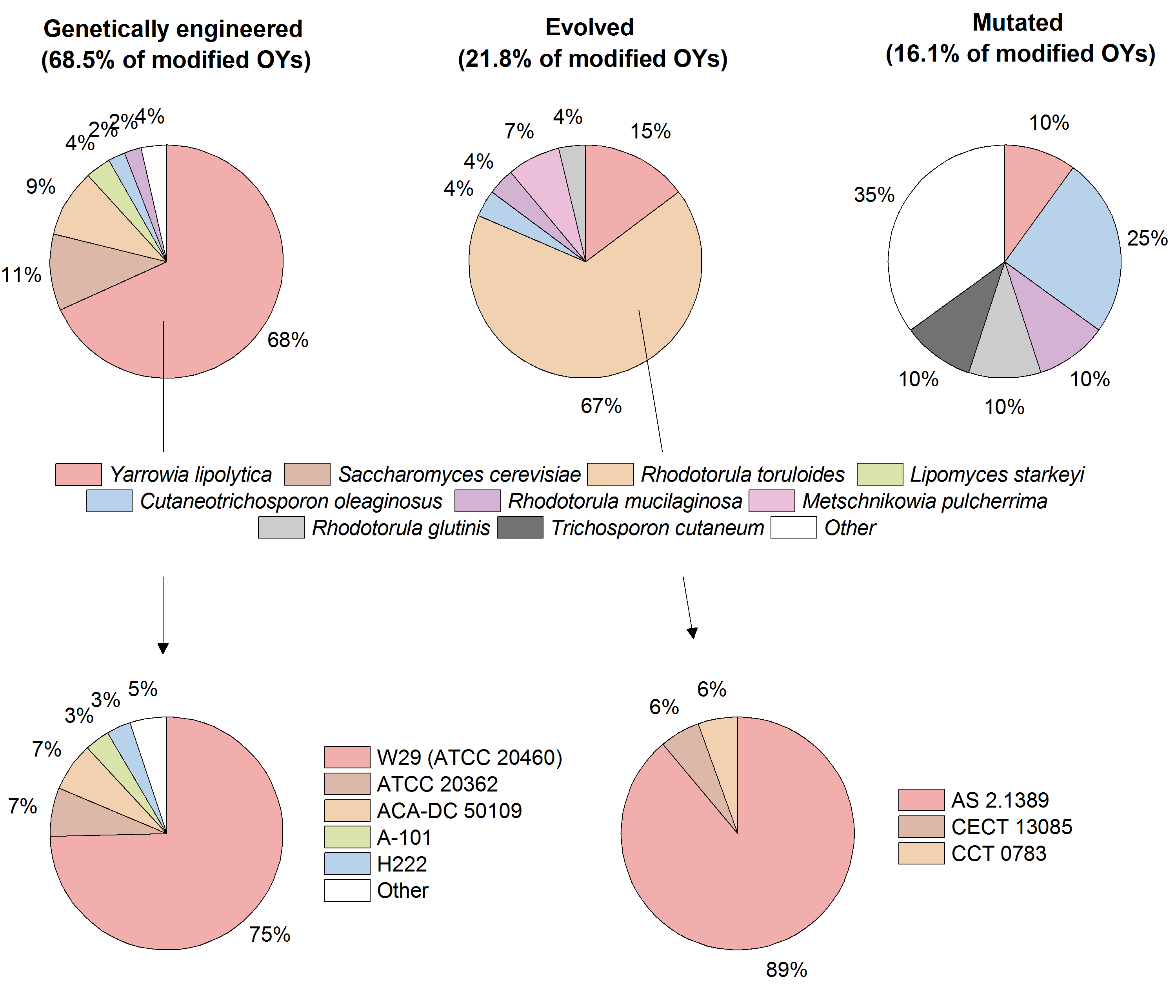
**Figure S6:** The oleaginous yeasts (OYs) most subjected to genetic modification (GM). Genetic modification took place via genetic engineering, evolution and/or mutation (double/triple counts possible if more than one technique was involved). Displayed is the percentage of a modified species used in relation to all OYs modified via the specified technique. For GM versions from the same wild type, only this with the highest lipid content was counted. For genetically engineered *Yarrowia lipolytica* and for *Rhodotorula toruloides*, the distributions of original wild-type strains are also displayed as percentage of a strain derivative used in relation to the species. The popular *Rhodotorula toruloides* strain Y4 was evolved from AS 2.1389. Culture collections are ACA-DC, Agricultural College of Athens - Dairy Collection (Greece); AS, China General Microbiological Culture Collection Center (China); ATCC, American Type Culture Collection (USA); CECT, Spanish Type Culture Collection (Spain); CCT, Tropical Culture Collection (Brazil). The full methodology used to collect and analyse the presented data is given at the beginning of this document.

# Figure S7


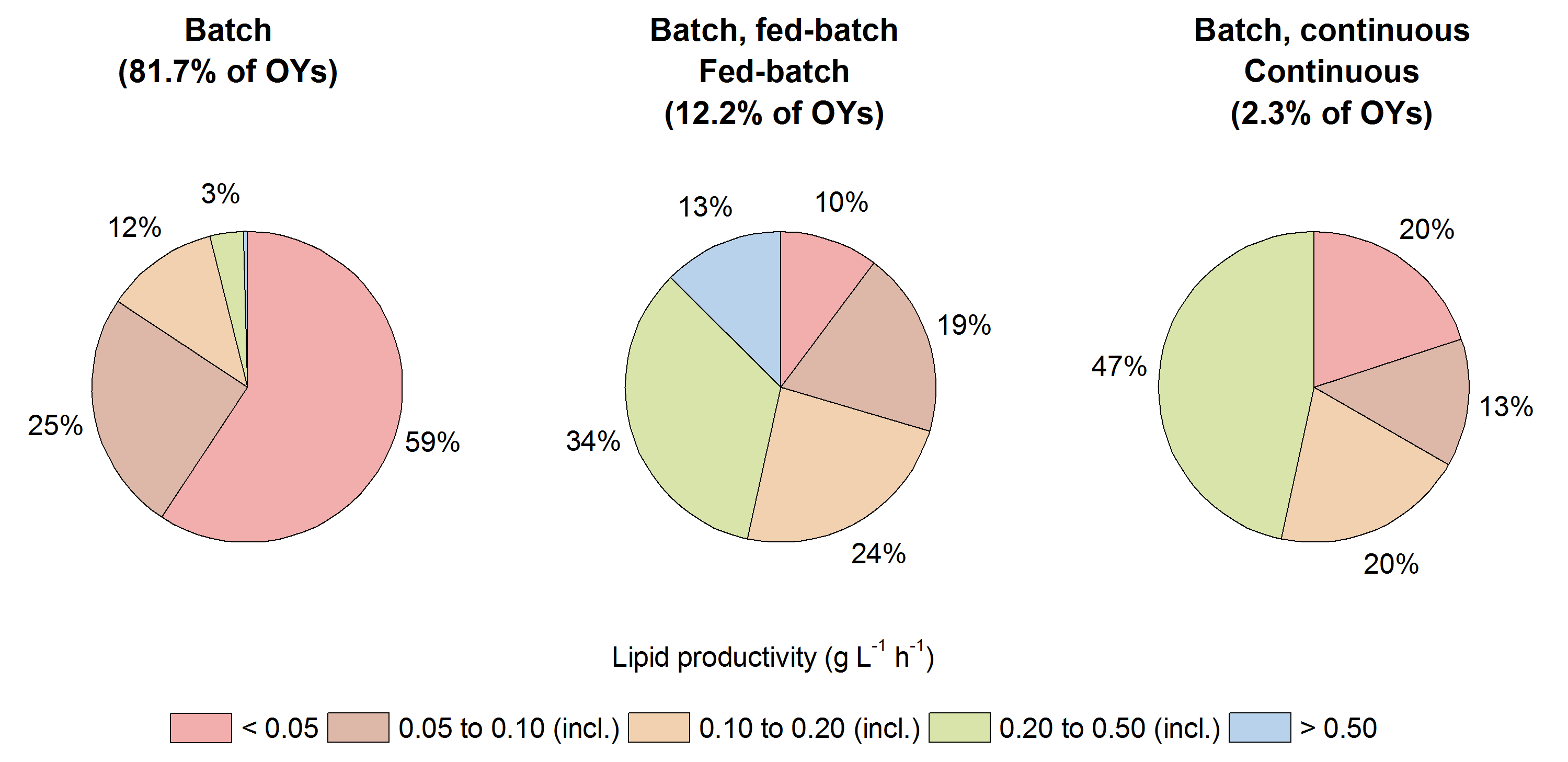


**Figure S7:** Lipid productivities in different operation modes. Per counted organism only highest productivity was recorded. The labels for values < 1% are not displayed. The full methodology used to collect and analyse the presented data is given at the beginning of this document.

# Figure S8
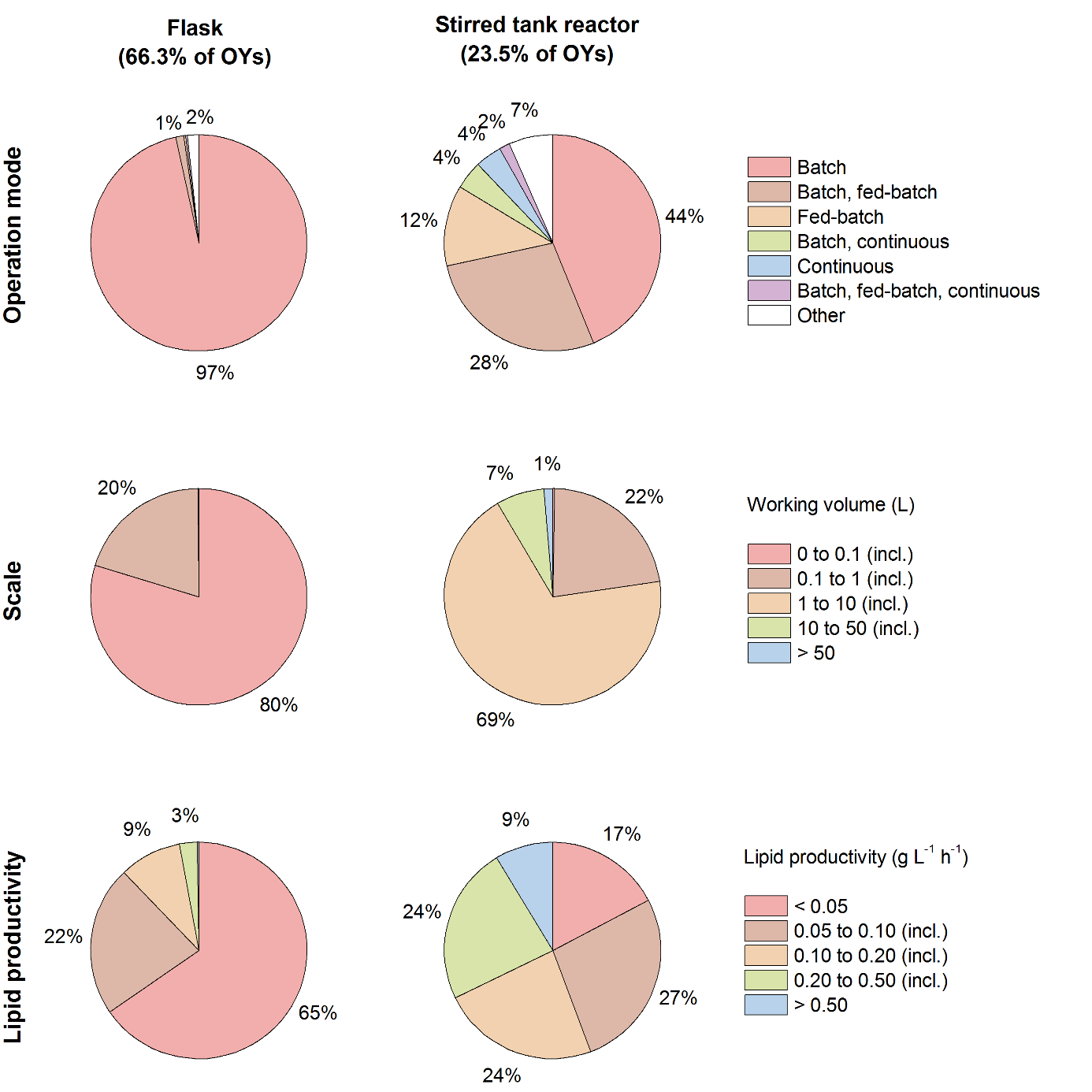


**Figure S8:** Differences in operation modes, scale and lipid productivity between flask and stirred tank reactor oleaginous yeast (OY) cultures. Displayed are the percentages of OY cultured in different operation modes, scales and with which productivity. Of all OYs, 93.8% have been cultured in either flasks or stirred tank reactors. Per counted organism only the largest vessel type and the highest productivity were recorded. It should be kept in mind, that often process development is conducted in flasks and the final process tested in stirred tank reactors. The labels for values < 1% are not displayed. The full methodology used to collect and analyse the presented data is given at the beginning of this document.

# Figure S9


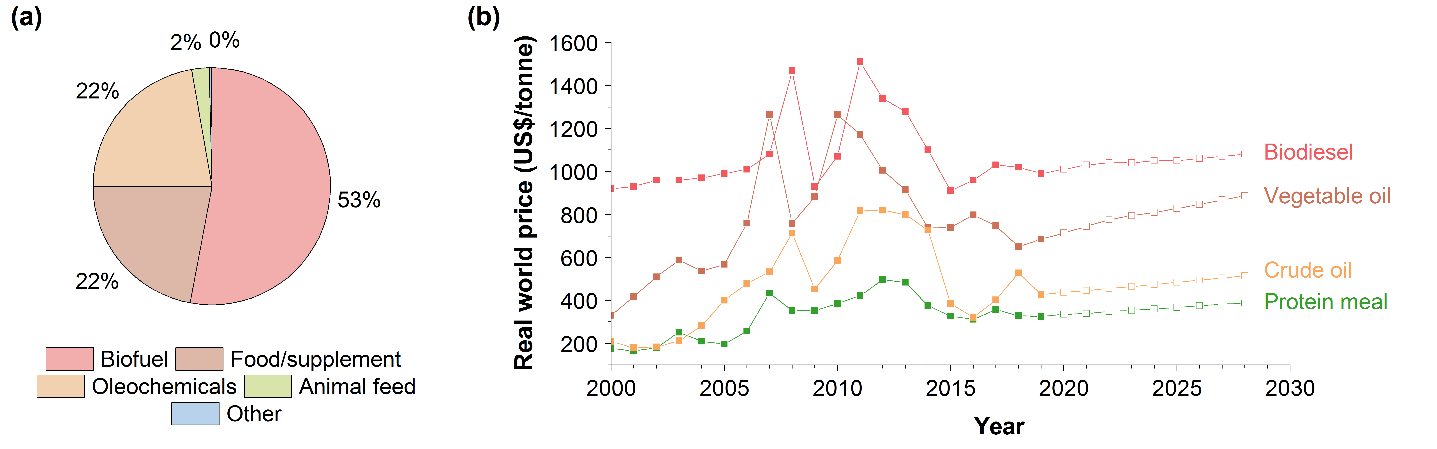


**Figure S9:** Applications of yeast lipids. (a) Proposed applications for yeast lipids as fraction of all those proposed in oleaginous yeast research and (b) the cost development of some of the potential commodity applications for yeast lipids (forecast from 2020) (27). In 89.1% of oleaginous yeast research publications one or more application has been proposed with an average of 1.8 proposed applications per publication. In the remaining 10.9% of publications no application has been proposed. The full methodology used to collect and analyse the presented data is given at the beginning of this document.

# Figure S10


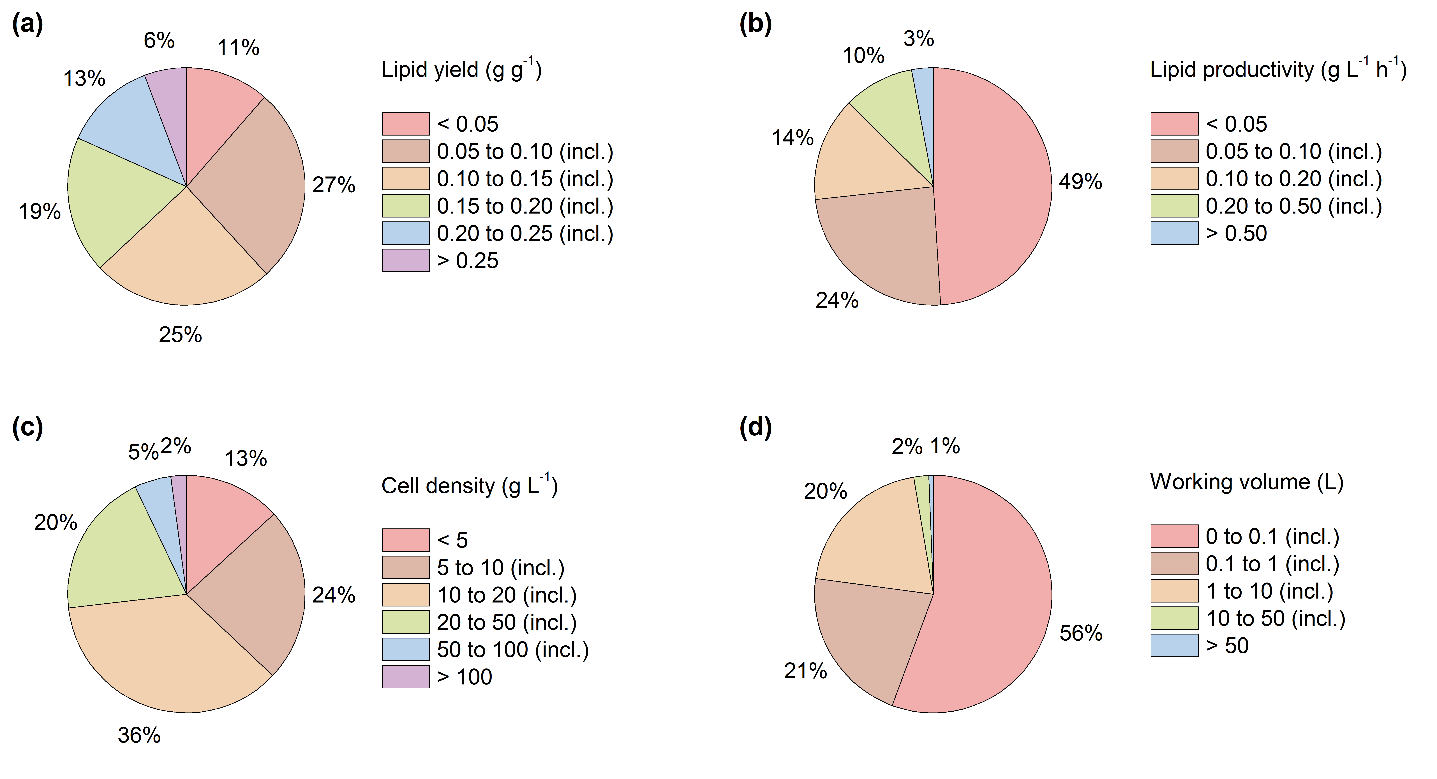


**Figure S10:** Economically relevant statistics of oleaginous yeast research. Displayed are the percentages of oleaginous yeasts (a) achieving a specific lipid yield on single saccharides; (b) achieving a specific lipid productivity; (c) cultured up to a specific cell density; and (d) cultured at a specific maximum cultivation scale. Per counted organism and publication, only the highest lipid yield, productivity, cell density and working volume were recorded. The full methodology used to collect and analyse the presented data is given at the beginning of this document.

# References

**References**

1. Thorpe RF, Ratledge C. Fatty acid distribution in triglycerides of yeasts grown on glucose or n-alkanes. J Gen Microbiol. 1972;72:151–63.

2. Woodbine M. Microbial fat: Microorganisms as potential fat producers. Prog Ind Microbiol. 1959;1:181–245.

3. Lipp M, Anklam E. Review of cocoa butter and alternative fats for use in chocolate - Part A. Compositional data. Food Chem. 1998;62(1):73–97.

4. Meesters PAEP, Huijberts GNM, Eggink G. High-cell-density cultivation of the lipid accumulating yeast Cryptococcus curvatus using glycerol as a carbon source. Appl Microbiol Biotechnol. 1996;45(5):575–9.

5. Chi Z, Zheng Y, Ma J, Chen S. Oleaginous yeast Cryptococcus curvatus culture with dark fermentation hydrogen production effluent as feedstock for microbial lipid production. Int J Hydrogen Energy. 2011;36(16):9542–50.

6. Zhang J, Fang X, Zhu XL, Li Y, Xu HP, Zhao BF, et al. Microbial lipid production by the oleaginous yeast Cryptococcus curvatus O3 grown in fed-batch culture. Biomass and Bioenergy. 2011;35(5):1906–11.

7. Capusoni C, Rodighiero V, Cucchetti D, Galafassi S, Bianchi D, Franzosi G, et al. Characterization of lipid accumulation and lipidome analysis in the oleaginous yeasts Rhodosporidium azoricum and Trichosporon oleaginosus. Bioresour Technol. 2017;238:281–9.

8. Lin J, Shen H, Tan H, Zhao X, Wu S, Hu C, et al. Lipid production by Lipomyces starkeyi cells in glucose solution without auxiliary nutrients. J Biotechnol. 2011;152(4):184–8.

9. Tsakona S, Kopsahelis N, Chatzifragkou A, Papanikolaou S, Kookos IK, Koutinas AA. Formulation of fermentation media from flour-rich waste streams for microbial lipid production by Lipomyces starkeyi. J Biotechnol. 2014;189:36–45.

10. Tamilalagan A, Singaram J. Oxidation stability of yeast biodiesel using Rancimat analysis: validation using infrared spectroscopy and gas chromatography–mass spectrometry. Environ Sci Pollut Res. 2019;26(3):3075–90.

11. Abeln F, Chuck CJ. Achieving a high-density oleaginous yeast culture: Comparison of four processing strategies using Metschnikowia pulcherrima. Biotechnol Bioeng. 2019;116:3200–14.

12. Lorenz E, Runge D, Marbà-Ardébol A-M, Schmacht M, Stahl U, Senz M. Systematic development of a two-stage fed-batch process for lipid accumulation in Rhodotorula glutinis. J Biotechnol. 2017;246:4–15.

13. Pan JG, Kwak MY, Rhee JS. High density cell culture of Rhodotorula glutinis using oxygen-enriched air. Biotechnol Lett. 1986;8(10):715–8.

14. Li Y, Zhao ZK, Bai F. High-density cultivation of oleaginous yeast Rhodosporidium toruloides Y4 in fed-batch culture. Enzyme Microb Technol. 2007;41(3):312–7.

15. Zhao X, Wu S, Hu C, Wang Q, Hua Y, Zhao ZK. Lipid production from Jerusalem artichoke by Rhodosporidium toruloides Y4. J Ind Microbiol Biotechnol. 2010;37:581–585.

16. Zhao X, Hu C, Wu S, Shen H, Zhao ZK. Lipid production by Rhodosporidium toruloides Y4 using different substrate feeding strategies. J Ind Microbiol Biotechnol. 2011;38(5):627–32.

17. Jin G, Yang F, Hu C, Shen H, Zhao ZK. Enzyme-assisted extraction of lipids directly from the culture of the oleaginous yeast Rhodosporidium toruloides. Bioresour Technol. 2012;111:378–82.

18. Babau M, Cescut J, Allouche Y, Lombaert-Valot I, Fillaudeau L, Uribelarrea J-L, et al. Towards a microbial production of fatty acids as precursors of biokerosene from glucose and xylose. Oil Gas Sci Technol. 2013;68(5):899–911.

19. Cescut J, Fillaudeau L, Molina-Jouve C, Uribelarrea JL. Carbon accumulation in Rhodotorula glutinis induced by nitrogen limitation. Biotechnol Biofuels. 2014;7(1):1–11.

20. Yang X, Jin G, Gong Z, Shen H, Bai F, Zhao ZK. Recycling microbial lipid production wastes to cultivate oleaginous yeasts. Bioresour Technol. 2015;175:91–6.

21. Zhang S, Ito M, Skerker JM, Arkin AP, Rao C V. Metabolic engineering of the oleaginous yeast Rhodosporidium toruloides IFO0880 for lipid overproduction during high-density fermentation. Appl Microbiol Biotechnol. 2016;100(21):9393–405.

22. Friedlander J, Tsakraklides V, Kamineni A, Greenhagen EH, Consiglio AL, MacEwen K, et al. Engineering of a high lipid producing Yarrowia lipolytica strain. Biotechnol Biofuels. 2016;9(77):1–12.

23. Qiao K, Wasylenko TM, Zhou K, Xu P, Stephanopoulos G. Lipid production in Yarrowia lipolytica is maximized by engineering cytosolic redox metabolism. Nat Biotechnol. 2017;35(2):173–7.

24. Xu J, Liu N, Qiao K, Vogg S, Stephanopoulos G. Application of metabolic controls for the maximization of lipid production in semicontinuous fermentation. Proc Natl Acad Sci. 2017;114(27):E5308–16.

25. Moon NJ, Hammond EG, Glatz BA. Conversion of cheese whey and whey permeate to oil and single-cell protein. J Dairy Sci. 1978;61(11):1537–47.

26. Davies JR. Scale up of yeast oil technology. In: Kyle DJ, Ratledge C, editors. Industrial applications of single cell oils. Urbana: American Oil Chemists Society; 1992.

27. OECD/FAO. OECD-FAO Agricultural Outlook (Edition 2019). OECD Agriculture Statistics (database). 2019.
